# Boosting Microglial Lipid Metabolism via TREM2 Signaling by Biomimetic Nanoparticles to Attenuate the Sevoflurane‐Induced Developmental Neurotoxicity

**DOI:** 10.1002/advs.202305989

**Published:** 2023-12-25

**Authors:** Wenting Li, Xiaowen Meng, Ke Peng, Yaobao Han, Hanghang Liu, Weiming Zhao, Gang Wang, Li Deng, Hong Liu, Zhen Li, Fuhai Ji

**Affiliations:** ^1^ Department of Anesthesiology the First Affiliated Hospital of Soochow University Suzhou Jiangsu 215006 China; ^2^ Center for Molecular Imaging and Nuclear Medicine State Key Laboratory of Radiation Medicine and Protection School for Radiological and Interdisciplinary Sciences (RAD‐X) Suzhou Medical College Soochow University Collaborative Innovation Center of Radiation Medicine of Jiangsu Higher Education Institutions Suzhou 215123 China; ^3^ Institute of Anesthesiology Soochow University Suzhou Jiangsu 215006 China; ^4^ Department of Anaesthesiology and Pain Medicine University of California Davis Health Sacramento CA 95817 USA

**Keywords:** biomimetic nanoparticles, lipid metabolism, neuroinflammation, sevoflurane‐induced neurotoxicity, TREM2

## Abstract

Lipid metabolism has been considered as a potential therapeutic target in sevoflurane‐induced neurotoxicity that can potentially affect the learning and memory function in the developmental brain. Recently, triggering receptor expressed on myeloid cells 2 (TREM2) is identified as a crucial step in regulating lipid metabolism and associated with the pathogenesis of neurodegenerative diseases. Herein, it is reported that quercetin modified Cu_2‐_ *_x_* Se (abbreviated as CSPQ) nanoparticles can ameliorate sevoflurane‐induced neurotoxicity by tuning the microglial lipid metabolism and promoting microglial M2‐like polarization via TREM2 signaling pathway, in which the apolipoprotein E (ApoE), and adenosine triphosphate‐binding cassette transporters (ABCA1 and ABCG1) levels are upregulated. Furthermore, the protective effects of CSPQ nanoparticles against sevoflurane‐induced neurotoxicity via TREM2 are further demonstrated by the small interfering RNA (siRNA)‐TREM2 transfected BV2 cells, which are obviously not influenced by CSPQ nanoparticles. The cell membrane coated CSPQ (referred as CSPQ@CM) nanoparticles can significantly reduce sevoflurane‐induced learning and memory deficits, improve lipid metabolism dysfunction, and promote the remyelination in the hippocampus of mice. The study shows great potential of targeting microglial lipid metabolism in promoting remyelination of neurons for treatment of neurotoxicity and neurodegenerative diseases.

## Graphical abstract

Lipid droplets as the components of myelin sheath and neuron cell membranes play an important role in the brain. Sevoflurane exposures in the neonatal can lead to developmental neurotoxicity through dysmyelination. Quercetin functionalized Cu_2‐_ *_x_* Se nanoparticles are used to modulate the lipid metabolism and polarization of microglia to improve the sevoflurane induced‐developmental neurotoxicity by targeting the surface TREM2.

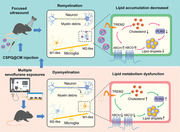

## 1 Introduction

Sevoflurane is a commonly used inhalational anesthetic agent for neonatal and pediatric patients due to the rapid onset of general anesthesia.^[^ ^1^ ^]^ However, clinical investigations have suggested that children three years old or younger with repeated exposure to inhalational anesthetics might have increasing risks of cognitive impairment and changes in their brain function.^[^ ^2^ ^]^ Similarly, preclinical studies have also shown that multiple sevoflurane exposures can induce neurobehavioral abnormalities in rodents, such as learning and memory deficits, anxiety related behaviors, and motor reflex deficits.^[^ ^3^ ^]^ Moreover, the U.S. Food and Drug Administration (FDA) has issued a drug safety communication warning stating that commonly used sedative and anesthetic medications have potential neurotoxic effects on children less than 3 years of age, and pregnant women during the third trimester underwent anesthesia for more than 3 h or repeatedly subjected to anesthetics.^[^ ^4^ ^]^ Therefore, it is crucial to effectively prevent the anesthetic agent induced neurotoxicity in these patients.

With the increasing concerns on the long‐term safety of neonatal multiple sevoflurane exposures, several studies have revealed that multiple sevoflurane anesthesia can induce the dysmyelination in the developmental brain.^[^ ^5^ ^]^ In the early postnatal period, multiple sevoflurane exposures can induce myelination defects and cognitive impairment in the young rhesus macaques and mice through reducing the expression of myelination related genes,^[^ ^6^ ^]^ indicating that the facilitation of remyelination is crucial for recovery of cognitive functions in sevoflurane‐induced developmental neurotoxicity. Importantly, the microglial lipid metabolism plays an important role in the process of remyelination, which involves the internalization of myelin debris, phagolysosome maturation and cholesterol recycling.^[^ ^7^ ^]^ Meanwhile, the large amounts of lipid‐rich myelin debris engulfed by microglia can not be effectively degraded and accumulated as lipid droplets,^[^ ^8^ ^]^ and the overload of lipid droplets in microglia significantly induced abnormal lipid metabolism. Some studies demonstrate that sevoflurane exposures could break the lipid homeostasis in the brain, which caused the impairment of nervous system.^[^ ^6^, ^9^ ^]^ However, there is no effective approach to regulate microglial lipid metabolism for promoting the remyelination of neurons in the multiple sevoflurane exposures induced developmental neurotoxicity.

It has been known that triggering receptor expressed on myeloid cells 2 (TREM2) is a single‐pass transmembrane immune receptor, which is selectively and highly expressed on microglia. TREM2 can be a multifaceted player in regulating the microglial function, including immune response,^[^ ^10^ ^]^ synaptic pruning,^[^ ^11^ ^]^ and phagocytosis activity.^[^ ^12^ ^]^ Intriguingly, it has also been identified as a microglial lipid‐sensor, because the activation of microglial TREM2 signaling can significantly accelerate clearance of myelin debris and promote remyelination in neurodegenerative diseases.^[^ ^13^ ^]^ In the process of lipid metabolism, the cholesterol recycling through adenosine triphosphate‐binding cassette (ABC) transporters ABCA1 and ABCG1 are mainly responsible for transporting lipid droplets out of microglia to maintain the normal physiological function.^[^ ^14^ ^]^ Moreover, the contribution of microglial TREM2 signaling to the lipid metabolism are mediated by cholesterol transporters ABCA1 and ABCG1.^[^ ^12^, ^13^ ^]^ These findings indicate that promoting the microglial lipid metabolism by modulating TREM2 signaling could be a novel strategy for treatment of cognitive dysfunction induced by multiple sevoflurane exposures, which has not been investigated.

Under the normal physiological circumstance, microglia are in a resting state and become activated in response to the endogenous or exogenous stimulation. The activated microglia mainly exist broadly in two different states, i.e., M1‐like microglia and M2‐like microglia.^[^ ^15^ ^]^ The activated M1‐like microglia are featured with the capability of producing inflammatory factors and reactive oxygen species (ROS). In contrast, M2‐like microglia protect the survival and plasticity of neurons by releasing anti‐inflammatory factors.^[^ ^16^ ^]^ It should be noted that sevoflurane exposure significantly promoted microglial M1‐like polarization and increased the generation of inflammatory factors.^[^ ^17^ ^]^ In addition, the previous work demonstrates that the overexpression of TREM2 by microglia obviously rescued cognitive impairment by promoting M2‐like polarization and inhibiting M1‐like polarization,^[^ ^18^ ^]^ suggesting that TREM2 as an important receptor not only regulates the lipid metabolism,^[^ ^12^, ^19^ ^]^ but also controls the microglia phenotypes to influence the pathogenesis of neurodegenerative diseases. In this context, selectively promoting TREM2‐mediated lipid metabolism and polarization of microglia into M2‐like phenotype could be an effective way to prevent sevoflurane‐induced developmental neurotoxicity and neurodegenerative diseases, although the mechanism remains unclear.

Quercetin (3,3′,4′,5,7‐pentahydroxyflavone; Qe) as the most common plant flavonoid is ubiquitously presented in various fruits, vegetables, and herbal medicines.^[^ ^20^ ^]^ It has been proposed to prevent the neurodegenerative diseases through its anti‐inflammatory, antioxidative and antiapoptotic properties.^[^ ^21^ ^]^ Nevertheless, the clinical application of Qe in the neurodegenerative diseases is limited due to its poor water solubility, chemical instability, low‐bioavailability, extensive metabolism, and difficulty in crossing the blood‐brain‐barrier (BBB). To overcome these issues, many new drug delivery systems have been studied and developed to improve its therapeutic effect.^[^ ^22^ ^]^ It's important to note that nanotechnology is playing a pivotal role in this aspect,^[^ ^23^ ^]^ and Qe can be coordinated with the surface atoms of nanoparticles to improve its water solubility, bioavailability and pharmacological activity.^[^ ^24^ ^]^ Among different types of nanoparticles, ultrasmall Cu_2‐_ *_x_* Se nanoparticles show advantages in crossing the BBB in comparison with larger counterparts.^[^ ^25^ ^]^ Our previous study demonstrates that our rationally designed biomimetic nanoparticles (abbreviated as CSPQ@CM), which are ultrasmall Cu_2‐_ *_x_* Se nanoparticles modified with Qe and coated with neuronal cell membrane (CM), can target microglial function and promote their M2‐like polarization for improving therapeutic efficacy of Parkinson's disease.^[^ ^26^ ^]^ However, the detail mechanism of microglial polarization mediated by CSPQ@CM nanoparticles remains unclear.

**Scheme 1:**
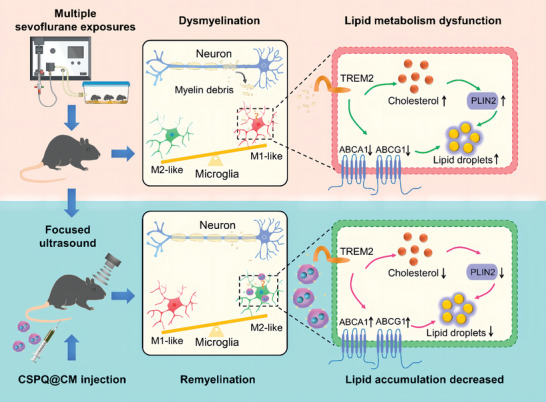
Biomimetic CSPQ@CM nanoparticles improved sevoflurane‐induced developmental neurotoxicity through targeting microglia to promote M2‐like polarization and decrease lipid accumulation via the TREM2 signaling for remyelination.

In this study, we aim to investigate the mechanism of polarization and lipid metabolism of microglia mediated by biomimetic CSPQ@CM nanoparticles, which can significantly improve the sevoflurane‐induced developmental neurotoxicity (**Scheme** **1**). We reveal that CSPQ@CM nanoparticles can target microglia to decrease lipid accumulation for promoting the remyelination of neurons, which was mediated by TREM2 signaling pathway. Our research demonstrates the great importance of targeting microglial lipid metabolism in remyelination of neurons for alleviation of neurotoxicity and treatment of neurodegenerative diseases.

## 2 Results and Discussion

### 2.1 Multiple Sevoflurane Exposures in Neonatal Period can Induce Cognitive Dysfunction in the Young Mice

**Figure 1:**
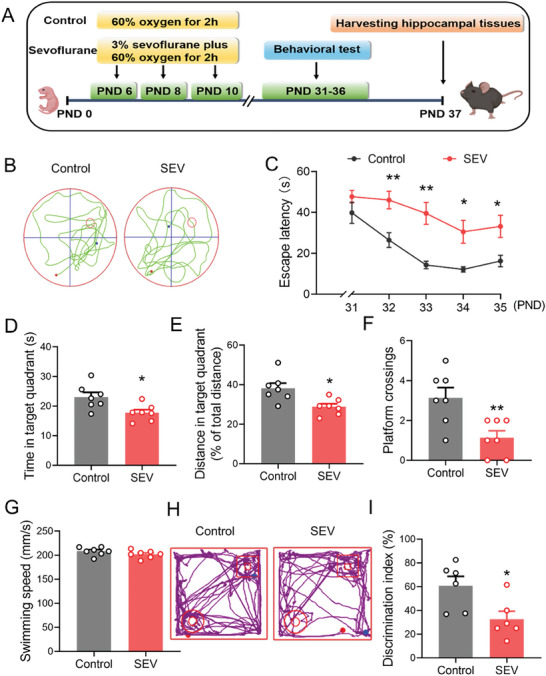
Multiple sevoflurane exposures induced cognitive dysfunction in young mice. A) Experimental protocol. B) Representative swimming traces of mice during Morris water maze (MWM) test with hidden platform, which was removed on the PND 36 (*n* = 7 mice for each group). C) Average escape latency of mice during the training phase in the MWM test. D) The time of mice spending in the target quadrant during the MWM test on the PND 36. E) Distance in the target quadrant during the MWM test on the PND 36. F) The frequency of mice crossing platform on the PND 36. G) Swimming speed. H) Representative motion track of mice in the novel‐object recognition (NOR) test from the Control group and sevoflurane (SEV) group (*n* = 6 mice for each group). I) The discrimination index of mice in the NOR test. The two‐sided unpaired Student's t‐test was used for comparison between two groups. Two‐way ANOVA with a Tukey post hoc analysis (C). Values were expressed as the mean ± SEM. *p < 0.05, **p < 0.01 versus Control group.

Multiple sevoflurane exposures in the neonatal period were reported to cause neurodegenerative changes in adolescent mice.^[^ ^27^ ^]^ To investigate the long‐term effect of multiple sevoflurane exposures in neonatal period on the cognitive function of young mice, neonatal mice were put in an anaesthetizing chamber filled with 3% sevoflurane for 2 h on the postnatal day (PND) 6, 8, and 10, respectively. The animal experimental scheme was presented in **Figure** **1**A. We first examined the spatial learning and memory of adolescent mice using the Morris water maze (MWM) test (Figure 1B). On the PND 31–35, the mice were trained to reach the platform for 5 days with 4 trials per day. The escape latency (time for mice to reach the platform) was recorded to evaluate their spatial learning. Our results show that the mice exposed to sevoflurane multiple times had longer escape latency than those from the Control group, suggesting an impaired spatial learning ability caused by the multiple sevoflurane exposures (Figure 1C). On the PND 36, the platform was removed to assess the memory function of mice. Compared to the mice from the Control group, the mice received multiple sevoflurane exposures failed to find the location of previous platform, spent less time in the target quadrant, had shorter distance in the target quadrant and lower frequency of crossing platform (Figure 1D–F), suggesting defects in their spatial memory ability. In contrast, the swimming speed of mice received multiple sevoflurane exposures was not significantly different from the mice in the Control group (Figure 1G).

Similarly, multiple exposures to sevoflurane in the neonatal period can induce cognitive impairment of young mice, as evidenced by the decreased discrimination index in the novel‐object recognition (NOR) test (Figure 1H,I). Additionally, the open field test was carried out to determine whether the multiple sevoflurane exposures‐induced cognitive deficits were resulted from the decreased spontaneous locomotor activity. The results show that no significant difference was observed in the time spent in the peripheral zone and the center zone, and transition number in the zone and mean speed between two groups (Figure S1A–E, Supporting Information). Taken together, these behavioral results indicate that multiple sevoflurane exposures can induce learning and memory impairment in the development of brain, but the damaged cognitive function is not associated with the reduced locomotor ability.

### 2.2 Multiple Sevoflurane Exposures can Lead to Abnormal Lipid Accumulation in Microglia in the Developmental Hippocampus of Mice

**Figure 2:**
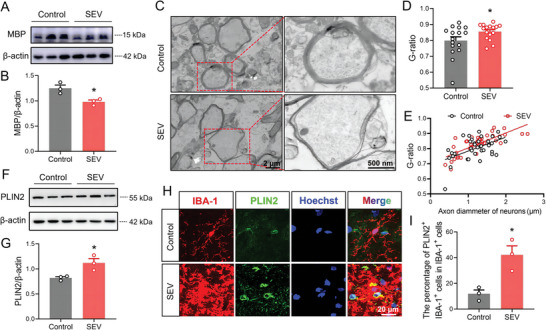
Multiple sevoflurane exposures led to abnormal lipid accumulation of microglia in the developmental hippocampus of mice. A) Western blot analysis of MBP in the hippocampus of mice from the Control group and SEV group. B) Sevoflurane decreased the MBP levels (*n* = 3 mice for each group). C) Representative images of TEM showing the ultra‐structure of myelin sheath in the CA1 region of hippocampus (scale bar = 2 µm; and 500 nm in the enlarged images). D) Quantification of G‐ratio representing the myelinated axons (*n* = 16 myelinated axons for each group). E) The efficiency index curves for axon caliber. F) Western blot analysis of PLIN2 in the hippocampus. G) Sevoflurane increased the PLIN2 protein levels (*n* = 3 mice for each group). H) Immunostaining images of PLIN2 in the hippocampus (*n* = 3 mice for each group, scale bar = 20 µm). I) Sevoflurane increased the abundance of PLIN2 in the microglia of the hippocampus. The two‐sided unpaired Student's *t*‐test was used for comparison between two groups. Values were expressed as the mean ± SEM. *p < 0.05 versus Control group.

The maintenance of cognitive function mainly depends on the normal physiological status of neurons.^[^ ^28^ ^]^ With the protective effect on neurons, the myelin sheath as the tubular membrane surrounding the axon plays an important role in maintaining the rapid propagation of nerve impulses, avoidance of interference between nerve impulses, and the regeneration of the axon.^[^ ^29^ ^]^ Therefore, the degradation of myelin is a prominent feature in the pathogenesis of neurological disorders involving cognitive dysfunction. Myelin basic protein (MBP) as one of the most abundant structural proteins in myelin is essential for myelin formation and compaction. Thus, we first examined the MBP level in the hippocampus tissues of mice on the PND 37 to identify the effects of multiple sevoflurane exposures on the myelin. The results show that MBP was significantly decreased in the mice exposed to sevoflurane (SEV group) compared to that of mice from the Control group (**Figure** **2**A,B). We also evaluated the ultra‐structure of myelin sheath in the CA1 region of hippocampus using transmission electron microscopy, which showed loosely wrapped myelin surrounding the axons in the mice from the SEV group, as compared to the compact myelin sheath observed in the Control group of mice (Figure 2C). To quantify dysmyelination, the thickness of myelin and diameter of axons were measured and used to calculate the myelin G‐ratio. The results show that the G‐ratio of myelin sheath in the hippocampus CA1 region was prominently increased in the mice received multiple sevoflurane exposures compared to that of mice from the Control group, suggesting that the myelin thickness was significantly decreased in the mice from the SEV group (Figure 2D). However, there was no significant difference in the axon caliber of myelin sheath among the mice from the SEV and Control groups (Figure 2E). These data are consistent with the previous study that multiple sevoflurane anesthesia in neonatal mice induced an obvious dysmyelination in the hippocampus of young mice,^[^ ^5^ ^]^ suggesting that the facilitation of remyelination in the hippocampus is crucial for improvement of the cognitive functions in sevoflurane‐induced developmental neurotoxicity.

As an important step for remyelination and tissue repair, the effective clearance of myelin debris is required in the region of dysmyelination in the brain. Microglia are responsible for the clearance of debris generated from damaged myelin sheath, and the facilitation of remyelination.^[^ ^30^ ^]^ During the progress of lipid metabolism, myelin debris are phagocytosed by microglia, then stored as cholesteryl esters and triacylglycerol in cytoplasmic lipid droplets. However, excessive cholesterol‐rich myelin debris may lead to the accumulation of lipid droplets in microglia in the dysmyelination lesions.^[^ ^31^ ^]^ In this study, the protein level of lipid droplets‐associated protein perilipin‐2 (PLIN2) in the hippocampus of mice on the PND37 was detected. The results show a significantly higher level of PLIN2 in the mice from the SEV group than that of mice in the Control group (Figure 2F,G). In addition, the immunostaining was carried out for detecting the co‐expression of PLIN2 and the microglial marker ionized calcium‐binding adaptor protein 1 (IBA‐1) in the hippocampus of mice on the PND37. The PLIN2 abundance was also significantly increased in the microglia of the hippocampus from the mice received multiple sevoflurane exposures (Figure 2H). The IBA‐1^+^ PLIN2^+^ cells in the SEV group were also more than those in the Control group (Figure 2I), indicating that multiple sevoflurane exposures led to the accumulation of lipid droplets in the microglia to inhibit the remyelination. The immunostaining of brain slices revealed a significant increase in the fluorescence intensity of IBA‐1^+^ microglia in the mice from the SEV group compared to the mice from the Control group (Figure S2A,B, Supporting Information), and the morphology analysis showed that the microglia in the SEV group displayed more amoeboid morphology. The results show that multiple sevoflurane exposures significantly increased the soma size of microglia, and decreased their total process lengthen and the number of branches (Figure S2C–E, Supporting Information). These findings demonstrate that abnormal lipid accumulation in the microglia is a key contributor to the neurodegenerative diseases.^[^ ^7^ ^]^

### 2.3 CSPQ Nanoparticles can Inhibit the Sevoflurane‐Induced Neuroinflammation by Promoting Microglial M2‐Like Polarization

Polarization of the pro‐inflammatory microglia into the anti‐inflammatory phenotype could promote remyelination,^[^ ^16^ ^]^ which suggests that anti‐inflammatory polarization of microglia is crucial for effective remyelination. Our previous study has demonstrated that CSPQ nanoparticles (Figure S3A, Supporting Information) can effectively scavenge reactive oxygen species (ROS) and promote microglial M2‐like polarization to reduce the H_2_O_2_‐induced neuroinflammation.^[^ ^26^ ^]^ In the present study, the effect of CSPQ nanoparticles on the physiological state of microglia was further investigated by using BV2 cells as a model. The BV2 cells were cultured with 10, 20, 40, 80, and 100 µm CSPQ nanoparticles, respectively, and their viability was measured by the CCK‐8 assay. The viability of BV2 cells was significantly decreased when the concentration of CSPQ nanoparticles was 100 µm, however, it slightly changed with the nanoparticle concentration increasing from 10, 20, 40, to 80 µm (Figure S3B, Supporting Information). Therefore, 80 µm CSPQ nanoparticles were selected to explore the nanoparticle protective effects on sevoflurane‐induced neurotoxicity in BV2 cells. Similar to the previous report, CSPQ nanoparticles obviously polarized BV2 cells into the M2‐like anti‐inflammatory phenotype (Figure S3C–H, Supporting Information).

**Figure 3:**
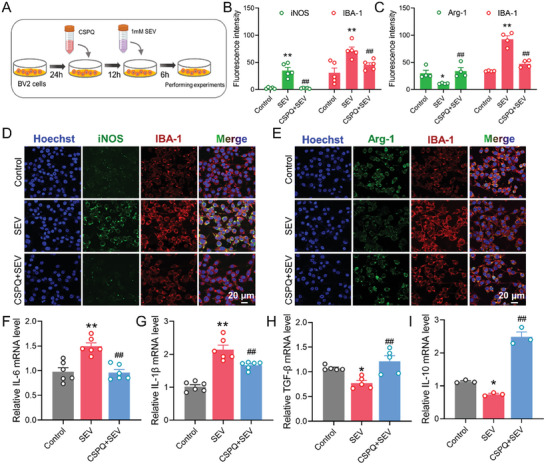
CSPQ nanoparticles inhibited sevoflurane‐induced neuroinflammation by promoting microglial M2‐like polarization. A) Experimental protocol for in vitro experiments. B,C) CSPQ nanoparticles reduced the upregulated iNOS expression and increased the downregulated Arg‐1 expression induced by sevoflurane in the BV2 cells (*n* = 4 or 5 wells for each group). D,E) Immunofluorescence images of iNOS and Arg‐1 in the BV2 cells (Scale bar = 20 µm). F,G) CSPQ nanoparticles inhibited the upregulation of mRNA levels of IL‐6 and IL‐1β induced by sevoflurane (*n* = 6 wells for each group). H,I) CSPQ nanoparticles enhanced the downregulated mRNA levels of TGF‐β and IL‐10 induced by sevoflurane (*n* = 3 or 5 wells for each group). The two‐sided one‐way ANOVA with a Tukey post hoc analysis was used for comparison among multiple groups. Values were expressed as the mean ± SEM. *p < 0.05, **p < 0.01 versus Control group; ^##^ *p* < 0.01 versus SEV group.

Sevoflurane exposures increased the M1‐like pro‐inflammatory phenotype and enhanced the release of inflammatory cytokines.^[^ ^32^ ^]^ To further demonstrate whether CSPQ nanoparticles could elicit the protective effects against sevoflurane‐induced neurotoxicity by promoting the polarization of microglia into anti‐inflammatory M2‐like phenotype. The cultured BV2 cells were randomly divided into Control group, SEV group and CSPQ+SEV group, respectively. BV2 cells in the Control group were cultured with fresh medium for 12 h and followed by culture with 0.1% DMSO for another 6 h. The cells in the SEV group were cultured with fresh medium for 12 h and followed by culture with 1 mm sevoflurane for another 6 h. BV2 cells in the CSPQ+SEV group were pretreated with 80 µm CSPQ nanoparticles for 12 h and followed by culture with 1 mm sevoflurane for another 6 h (**Figure** **3**A). According to the biomarkers of activated microglia,^[^ ^15^ ^]^ we verified BV2 cells to be classical M1‐like phenotype (pro‐inflammatory) and the alternative M2‐like phenotype (anti‐inflammatory) by the immunofluorescence staining. The results indicate that sevoflurane exposures significantly increased the M1‐like phenotypic marker inducible nitric oxide synthase (iNOS), and decreased M2‐like phenotypic marker arginase‐1 (Arg‐1) in BV2 cells, which was reversed by pretreatment with 80 µm CSPQ nanoparticles (Figure 3B–E). Additionally, sevoflurane treatment enhanced the production of pro‐inflammatory mediators, including interleukin‐6 (IL‐6) and interleukin‐1β (IL‐1β), whereas inhibited the expression of anti‐inflammatory mediators, including interleukin‐10 (IL‐10) and transforming growth factor‐β (TGF‐β). Moreover, pretreatment of sevoflurane stimulated BV2 cells with CSPQ nanoparticles significantly decreased the production of pro‐inflammatory mediators, and increased the expression of anti‐inflammatory mediators (Figure 3F–I). Therefore, CSPQ nanoparticles can reduce the sevoflurane‐induced neuroinflammation by promoting the polarization of M1‐like microglia into M2‐like microglia.

### 2.4 CSPQ Nanoparticles can Alleviate Sevoflurane‐Induced Lipid Accumulation by Increasing Microglial Cholesterol Efflux

**Figure 4:**
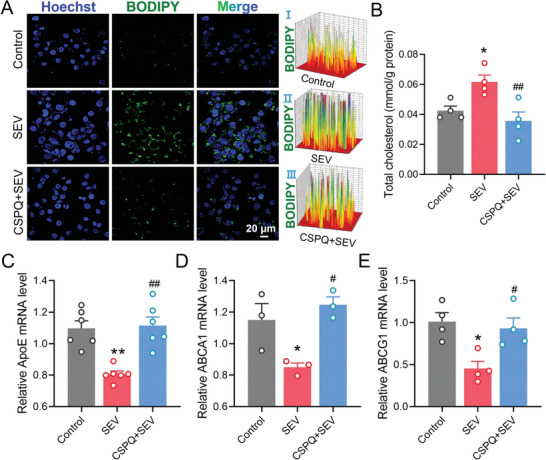
CSPQ nanoparticles alleviated sevoflurane‐induced lipid accumulation by increasing microglial cholesterol efflux. A) Immunofluorescence images of BV2 cells for detecting the intracellular accumulation of lipid droplets, and CSPQ nanoparticles reduced the accumulation of lipid droplets induced by sevoflurane (*n* = 4 wells for each group, blue: cell nuclei stained by Hoechst 33 342 dye; green: BODIPY, scale bar = 20 µm). The fluorescence distribution and intensity in BV2 cells were shown in I‐III. B) CSPQ nanoparticles inhibited the increased the intracellular cholesterol level induced by sevoflurane (*n* = 4 wells for each group). C‐E) CSPQ nanoparticles increased the downregulated mRNA expression of ApoE, ABCA1, and ABCG1 induced by sevoflurane (*n* = 3 – 6 wells for each group). The two‐sided one‐way ANOVA with a Tukey post hoc analysis was used for comparison among multiple groups. Values were expressed as the mean ± SEM. *p < 0.05, **p< 0.01 versus Control group; ^##^ *p* < 0.01 versus SEV group.

It has been demonstrated that Qe can regulate lipid metabolism‐related genes to alleviate kidney lipid accumulation in streptozotocin‐induced diabetic nephropathy rats.^[^ ^33^ ^]^ It can also prevent lipid accumulation in RAW264.7 macrophages via the endoplasmic reticulum (ER) stress pathway.^[^ ^34^ ^]^ The accumulation of lipid droplets in microglia represents a dysfunctional and pro‐inflammatory state in the brain of neurodegenerative disease.^[^ ^35^ ^]^ To investigate whether CSPQ nanoparticles could reduce lipid accumulation, the level of lipid droplets in BV2 cells was measured by BODIPY 493/503 staining. Compared to the cells from the SEV group, CSPQ nanoparticles significantly decreased the accumulation of lipid droplets in BV2 cells (**Figure** **4**A). Meanwhile, we examined the cholesterol level in BV2 cells using the intracellular cholesterol content analysis kit, and found that CSPQ nanoparticles also significantly reduced the intracellular total cholesterol level (Figure 4B).

Since acyl‐CoA:cholesterol acyltransferase 1 and 2 (ACAT1 and ACAT2) are mainly responsible for converting cholesterol to cholesteryl esters that stored in microglia as cytosolic lipid droplets,^[^ ^14^ ^]^ we first detected the mRNA levels of cholesterol acyltransferases. The results show that sevoflurane did not alter the ACAT1 and ACAT2 levels in BV2 cells (Figure S4A,B, Supporting Information). Next, we determined the mRNA level of cholesterol transporter apolipoprotein E (ApoE) and its binding partners ABCA1 and ABCG1 in BV2 cells, since they also play a crucial role in preventing intracellular lipid accumulation through regulating cholesterol efflux from microglia. The results clearly show that sevoflurane significantly reduced the mRNA expression of ApoE, ABCA1, and ABCG1 in BV2 cells, which were recovered to the normal levels by CSPQ nanoparticles (Figure 4C–E). Therefore, CSPQ nanoparticles can promote the cholesterol efflux from microglia influenced by sevoflurane.

Our previous work demonstrates that CSPQ nanoparticles can efficiently eliminate ROS and polarize microglia into anti‐inflammatory M2 phenotype.^[^ ^26^ ^]^ Therefore, we further investigated the relationship between lipid metabolism and microglial polarization. BV2 cells were treated with 1 µg mL^−1^ lipopolysaccharide (LPS) and 25 ng mL^−1^ interleukin‐4 （IL‐4）to induce the M1 pro‐inflammatory phenotype and M2‐like anti‐inflammatory phenotype, respectively. The results show that M1‐like microglia significantly inhibited the lipid metabolism, on the contrary, M2‐like microglia remarkedly accelerated the lipid metabolism (Figure S5A–E, Supporting Information), indicating that targeting lipid metabolism by promoting microglial M2‐like polarization could improve the outcome of metabolic dysfunction associated diseases. These data prove that CSPQ nanoparticles can promote the lipid metabolism and reduce the accumulation of lipid droplets in microglia by their mediated polarization. In addition, the lipid droplets overloaded in microglia significantly increased the reactive oxygen species (ROS) accumulation, resulting in the inhibition of remyelination in the neurodegenerative diseases.^[^ ^7^, ^29^ ^]^ Our CSPQ nanoparticles can protect BV2 cells against the sevoflurane‐induced impairment by scavenging ROS (Figure S6A, Supporting Information) and enhancing the phagocytosis ability of microglia (Figure S6B–D, Supporting Information).

### 2.5 Knockdown of TREM2 can Significantly Abolish CSPQ Nanoparticles’ Protective Effect on Sevoflurane Induced Neuroinflammation and Lipid Accumulation in Microglia

**Figure 5:**
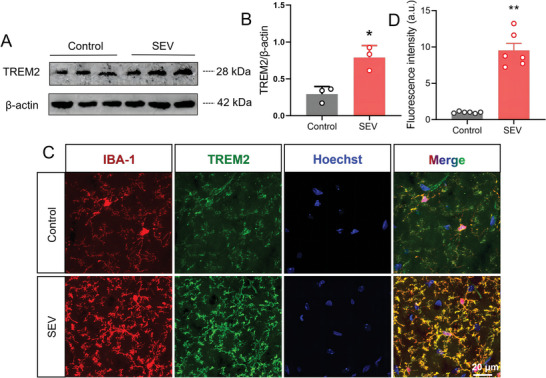
Multiple sevoflurane exposures upregulated the TREM2 expression in the microglia from the hippocampus of mice. A) Western blot analysis of TREM2 in the hippocampus. B) Multiple sevoflurane exposures increased the level of TREM2 protein in the hippocampus (*n* = 3 mice for each group). C,D) Multiple sevoflurane exposures enhanced the co‐expression of IBA‐1 and TREM2 in microglia from the hippocampus (*n* = 6 mice for each group, scale bar = 20 µm). The two‐sided unpaired Student's *t*‐test was used for comparison between two groups. Values were expressed as the mean ± SEM. *p < 0.05 versus Control group.

Based on the above results, we investigated the molecular mechanism of CSPQ nanoparticles eliciting the protective effect on the sevoflurane‐induced developmental neurotoxicity. TREM2, as the lipid‐sensor specifically expressed by microglia, has been demonstrated to enhance microglial survival, proliferation, phagocytosis, and lipid metabolism.^[^ ^36^ ^]^ In the differentiation trajectory of microglia, our recent study has demonstrated that multiple sevoflurane exposures significantly increased the proportion of subcluster with expressing P2Y12 receptor (P2ry12), C‐X3‐C motif chemokine receptor 1 (Cx3cr1), and TREM2, and this subcluster was related to sensing cellular injury, myelin debris clearance and promotion of inflammation.^[^ ^37^ ^]^ We detected the TREM2 protein level in the hippocampus of mice on the PND 37, and found that multiple sevoflurane exposures significantly increased the expression of TREM2 protein (**Figure** **5**A,B). Furthermore, we investigated the effects of multiple sevoflurane exposures on the co‐expression of TREM2 and IBA‐1 in the hippocampus of mice. Compared to the mice from the Control group, the fluorescence intensity of TREM2 in microglia from the hippocampus of mice in the SEV group was significantly increased (Figure 5C,D), indicating that up‐regulated TREM2 expression in microglia may represent an adaptive mechanism in response to the sevoflurane‐induced neurotoxicity. We also found that CSPQ nanoparticles significantly enhanced the TREM2 protein expression in the normal cultured BV2 cells (Figure S7A,B, Supporting Information). These results indicate that the protective effect of CSPQ nanoparticles on sevoflurane‐induced neurotoxicity could be mediated by TREM2 signaling in microglia.

To further demonstrate the important role of TREM2 signaling in microglia, we transfected BV2 cells with three different TREM2 siRNA (siRNA1, siRNA2, and siRNA3). Transfection with the samll interfering RNAs (siRNA) significantly decreased TREM2 protein levels compared to the Control group or negative group of cells, and the siRNA3 was the most efficient one to decrease the TREM2 protein expression, which was chosen for the following experiments (Figure S7C,D, Supporting Information). The siRNA‐TREM2 transfection obviously inhibited the mRNA expression of TREM2 in BV2 cells treated with sevoflurane and CSPQ nanoparticles (Figure S7E, Supporting Information). Additionally, the effect of si‐TREM2 alone on the viability of BV2 cells was investigated. The results show that si‐TREM2 transfection did not alter the viability of BV2 cells on the physiological state (Figure S7F, Supporting Information).

**Figure 6:**
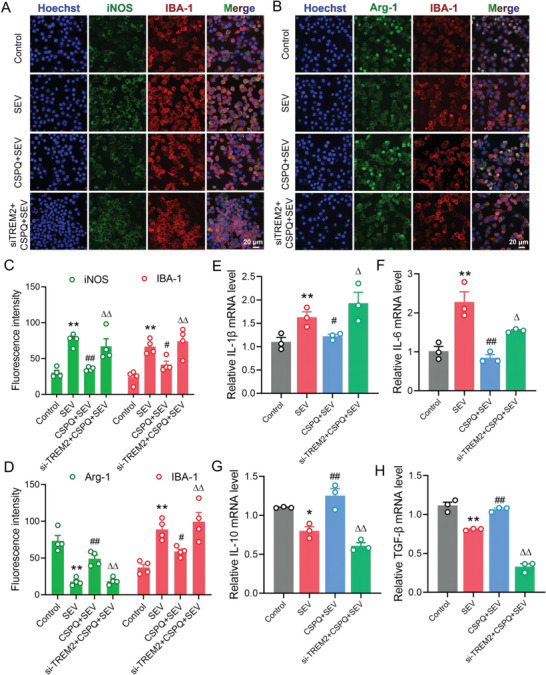
Knockdown of TREM2 gene significantly abolished the protective effects of CSPQ nanoparticles by inhibiting microglial M2‐like polarization. A) Immunofluorescence images of iNOS and IBA‐1 in BV2 cells (Scale bar = 20 µm). B) Immunofluorescence images of Arg‐1 and IBA‐1 in BV2 cells (Scale bar = 20 µm). C,D) The siRNA‐TREM2 transfection increased iNOS expression and decreased Arg‐1 expression (*n* = 4 wells for each group). E,F) The siRNA‐TREM2 transfection enhanced IL‐1β and IL‐6 levels (*n* = 3 wells for each group). G,H) The siRNA‐TREM2 transfection reduced IL‐10 and TGF‐β levels (*n* = 3 wells for each group). The two‐sided one‐way ANOVA with a Tukey post hoc analysis was used for comparison among multiple groups. Values were expressed as the mean ± SEM. *p < 0.05, **p< 0.01 versus Control group; ^#^ *p* < 0.05, ^##^ *p* < 0.01 versus SEV group; ^∆^ *p* < 0.05, ^∆∆^ *p* < 0.01 versus CSPQ + SEV group.

Subsequently, we investigated whether TREM2 knockdown could alter the protective effect of CSPQ nanoparticles against sevoflurane‐induced neuroinflammation. The immunofluorescence showed that siRNA‐TREM2 transfection significantly increased iNOS and decreased Arg‐1 expressions in BV2 cells when compared to those pretreated with CSPQ nanoparticles during sevoflurane‐induced neurotoxicity (**Figure** **6**A–D). Similarly, siRNA‐TREM2 transfection also increased the pro‐inflammatory mediators (IL‐1β and IL‐6) expressions (Figure 6E,F), as well as decreased anti‐inflammatory mediators (IL‐10 and TGF‐β) levels in BV2 cells pretreated with CSPQ nanoparticles during sevoflurane‐induced neuroinflammation (Figure 6G,H). Therefore, TREM2 knockdown can partly abolish the protective role of CSPQ nanoparticles against sevoflurane‐induced neuroinflammation by inhibiting microglial M2‐like polarization.

**Figure 7:**
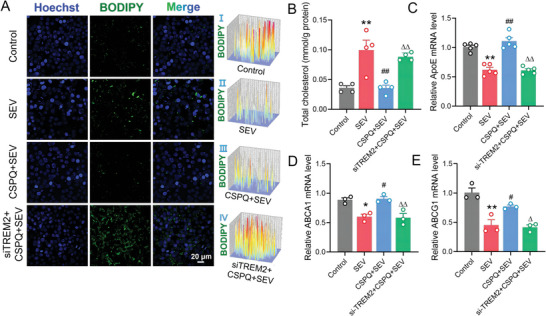
Knockdown of TREM2 gene significantly blocked the protective effects of CSPQ nanoparticles by increasing microglial lipid metabolism. A) Immunofluorescence images of BV2 cells for detecting the intracellular accumulated lipid droplets, and siRNA‐TREM2 transfection increased the accumulation of lipid droplets in BV2 cells pretreated with CSPQ nanoparticles (blue: cell nuclei stained by Hoechst 33 342 dye; green: BODIPY, scale bar = 20 µm). The fluorescence distribution and intensity in BV2 cells were shown in I‐III. B) The siRNA‐TREM2 transfection increased intracellular cholesterol level (n = 4 wells for each group). C–E) The siRNA‐TREM2 transfection reduced mRNA expressions of ApoE, ABCA1 and ABCG1 in BV2 cells (*n* = 3 or 5 wells for each group). The two‐sided one‐way ANOVA with a Tukey post hoc analysis was used for comparison among multiple groups. Values were expressed as the mean ± SEM. *p < 0.05, **p< 0.01 versus Control group; ^#^ *p* < 0.05, ^##^ *p* < 0.01 versus SEV group; ^∆^ *p* < 0.05, ^∆∆^ *p* < 0.01 versus CSPQ + SEV group.

The si‐TREM2 transfection also increased the accumulation of lipid droplets and intracellular total cholesterol level in BV2 cells pretreated with CSPQ nanoparticles during sevoflurane‐induced abnormal lipid metabolism (**Figure** **7**A,B). Besides, siRNA‐TREM2 transfection reduced the mRNA expressions of ApoE, ABCA1, and ABCG1 in BV2 cells pretreated with CSPQ nanoparticles during sevoflurane‐induced abnormal lipid metabolism (Figure 7C–E). These results indicate that siRNA‐TREM2 transfection significantly blocked the protective effect of CSPQ nanoparticles against sevoflurane‐induced abnormal lipid metabolism in BV2 cells. The accumulation of lipid droplets induced by TREM2 knockdown proves that the protective role of CSPQ nanoparticles against sevoflurane‐induced abnormal lipid metabolism was mediated by TREM2 signaling.

### 2.6 CSPQ@CM Nanoparticles can Improve the Cognitive Deficits in Young Mice Induced by Multiple Sevoflurane Exposures in their Neonatal Period

**Figure 8:**
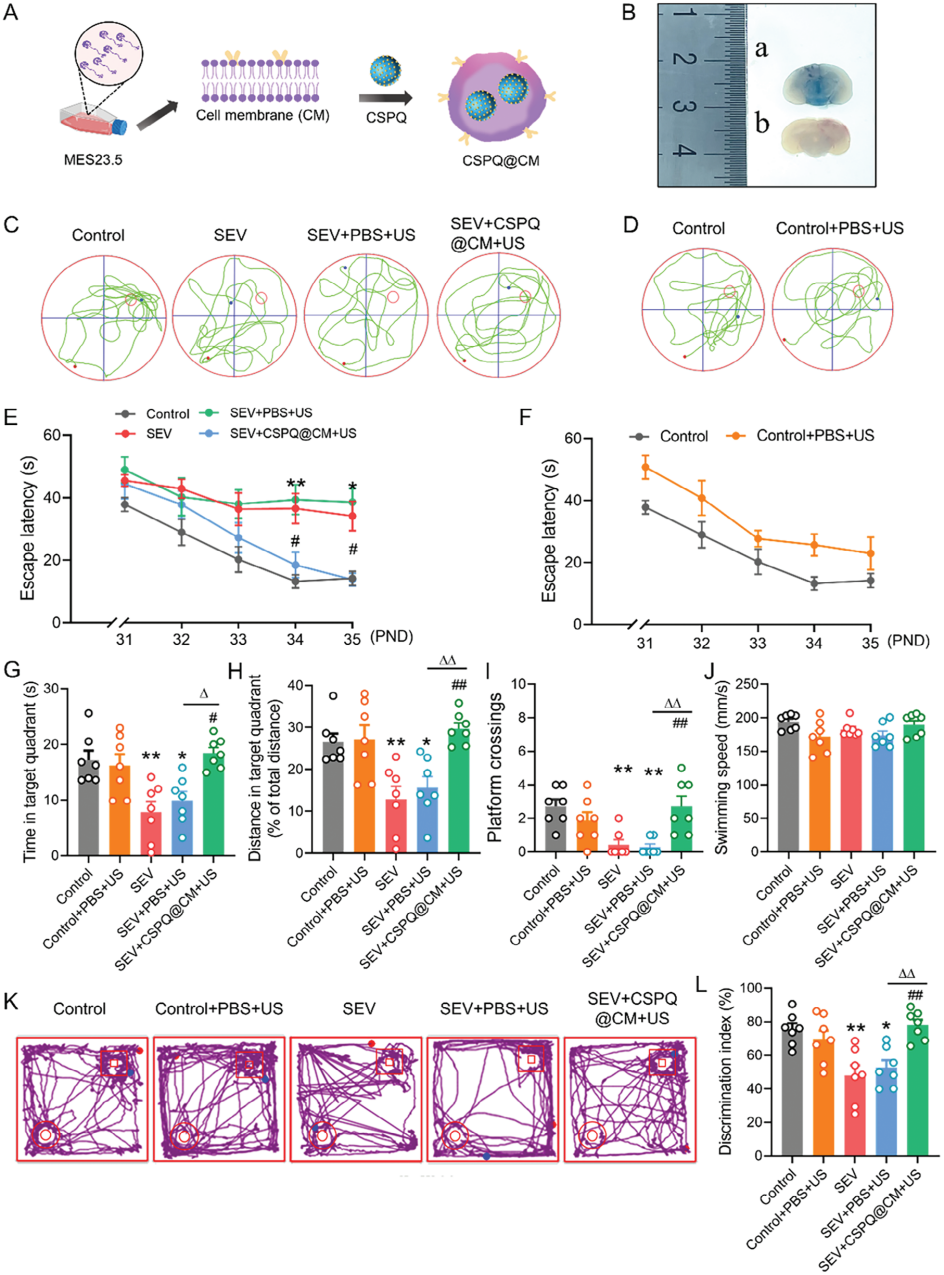
CSPQ@CM nanoparticles improved the cognitive deficits in young mice induced by multiple sevoflurane exposures in their neonatal period. A) CSPQ nanoparticles were coated with MES23.5 cell membrane. B) Evans blue staining of mouse brain slices. C,E) Treatment of mice with CSPQ@CM nanoparticles significantly decreased the elevated escape latency induced by multiple sevoflurane exposures (*n* = 7 mice for each group). D,F) PBS injection and treatment with the focused ultrasound have no significant effect on the escape latency in the healthy Control mice (*n* = 7 mice for each group). G) The time of mice spending in the target quadrant during MWM test on the PND 36 (*n* = 7 mice for each group). H) Distance in the target quadrant during the MWM test on the PND 36 (*n* = 7 mice for each group). I) The frequency of mice crossing the platform during the MWM test on the PND 36 (*n* = 7 mice for each group). J) The swimming speed during the MWM test on the PND 36 (*n* = 7 mice for each group). K) Representative motion track of the NOR test. L) The discrimination index of the NOR test (*n* = 7 mice for each group). The two‐sided one‐way ANOVA with a Tukey post hoc analysis was used for comparison among multiple groups. Two‐way ANOVA with a Tukey post hoc analysis (E, F). Values were expressed as the mean ± SEM. *p < 0.05, **p< 0.01 versus Control group; ^#^ *p* < 0.05, ^##^ *p* < 0.01 versus SEV group; ^∆^ *p* < 0.05, ^∆∆^ *p* < 0.01 versus SEV + PBS + US group.

The ultrasmall Cu_2‐_ *_x_* Se nanoparticles show advantages in crossing the blood‐brain barrier (BBB) in comparison with larger counterparts and can be efficiently delivered to the brain tissue under the assistance of focused ultrasound.^[^ ^25^, ^38^ ^]^ To endow CSPQ nanoparticles with targeting capability, they were coated with MES23.5 cell membrane (**Figure** **8**A). The resultant CSPQ@CM nanoparticles were intravenously injected into the mice, followed by transient opening of the BBB with focused ultrasound (1.0 MHz, 90s). Evans blue (2% wt) was used to examine the opening of the blood brain barrier (BBB) after perfusion (Figure 8B). The rubeanic acid (RA) staining clearly showed the copper stains in the hippocampus, which illustrateed the deposition of nanoparticles in the brain (Figure S8, Supporting Information). Our previous study has demonstrated that CSPQ nanoparticles can improve the cognitive function by targeting the microglia in the mice model of Parkinson's disease.^[^ ^26^ ^]^ In the current work, we mainly further investigated the neuroprotective of the CSPQ@CM on sevoflurane‐induced cognitive deficits in the developmental brain, and the mice were randomly divided into five groups: (I) normal healthy mice (Control group); (II) normal healthy mice intravenously injected with phosphate buffered saline (PBS) followed by sonication with focused ultrasound (Control +PBS+US group); (III) the mice received multiple sevoflurane exposures (SEV group); (IV) the mice in sevoflurane group injected with PBS followed by sonication with focused ultrasound (SEV+PBS+US group); (V) the mice in sevoflurane group injected with CSPQ@CM nanoparticles followed by sonication with focused ultrasound (SEV+CSPQ@CM+US group). Animals were intravenously administrated with the CSPQ@CM nanoparticles at a dose of 12 mg kg^−1^ body weight at two different time points. The first injection time was on the PND 27, and the second injection time was on the PND 29. In the training phase of mice on the PND 31–35, treatment with CSPQ@CM nanoparticles significantly decreased their elevated escape latency induced by multiple sevoflurane exposures (Figure 8C,E). In addition, the PBS injection and the focused ultrasound treatment had no significant effect on the escape latency of healthy mice in the Control group (Figure 8D,F). Compared to the mice in the SEV+PBS+US group, the SEV+ CSPQ@CM+US group of mice obviously spent more time in the target quadrant, had longer distance in the target quadrant and higher frequency of crossing platform in the testing phase on the PND 36 (Figure 8G–I). The swimming speed of mice in the SEV+CSPQ@CM+US group was not significantly different from the SEV+PBS+US group of mice (Figure 8J). In the NOR test, treatment of mice with CSPQ@CM nanoparticles significantly reversed the decreased discrimination index induced by multiple sevoflurane exposures (Figure 8K,L). Additionally, no significant difference was observed in the time spent in peripheral zone and in total zone, transition number in the zone and mean speed among these groups of mice (Figure S9A–E, Supporting Information). These results demonstrate that the neuroprotective CSPQ@CM nanoparticles against sevoflurane‐induced cognitive deficits was mainly related to the improvement of learning and memory capability.

### 2.7 CSPQ@CM Nanoparticles can Alleviate Microglial Lipid Accumulation and Promote Remyelination in the Developmental Hippocampus of Young Mice

**Figure 9:**
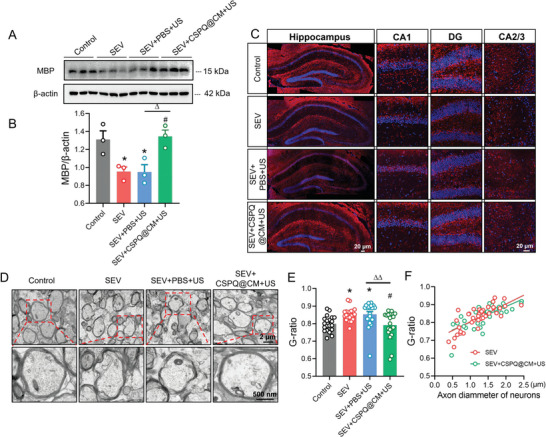
CSPQ@CM nanoparticles promoted the remyelination in the hippocampus of young mice induced by multiple sevoflurane exposures in their neonatal period. A,B) CSPQ@CM nanoparticles increased the MBP protein levels (*n* = 3 mice for each group). C) CSPQ@CM nanoparticles increased the fluorescence intensity of MBP in the hippocampus of CA1 region (Scale bar = 20 µm). D) Representative TEM images showing the ultra‐structure of myelin sheath in the CA1 region of hippocampus (scale bar = 2 µm; and 500 nm in the enlarged insert). E) Quantification of G‐ratio representing the myelinated axons (*n* = 20 myelinated axons for each group). F) The efficiency index curves for axon caliber. The two‐sided one‐way ANOVA with a Tukey post hoc analysis was used for comparison among multiple groups. Values were expressed as the mean ± SEM. *p < 0.05 versus Control group; ^#^ *p* < 0.05 versus SEV group; ^∆^ *p* < 0.05, ^∆∆^ *p* < 0.01 versus SEV+PBS+US group.

After the above behavioral test, we further investigated the therapeutic effects of CSPQ@CM nanoparticles on the dysmyelination in the hippocampus of the mice received multiple sevoflurane exposures. Compared to the mice in the SEV+PBS+US group, the MBP levels were significantly increased in the hippocampus of mice from the SEV+CSPQ@CM+US group on the PND 37 (**Figure** **9**A–C). Meanwhile, we further evaluated the ultra‐structure of myelin sheath in the CA1 region of mouse hippocampus (Figure 9D), and found that CSPQ@CM nanoparticles significantly blocked the decrease of myelin thickness in the young mice induced by multiple sevoflurane exposures in their neonatal period (Figure 9E). But there was no significant difference in the axon caliber of myelin sheath between the SEV+CSPQ@CM+US group and SEV+PBS+US group (Figure 9F).

**Figure 10:**
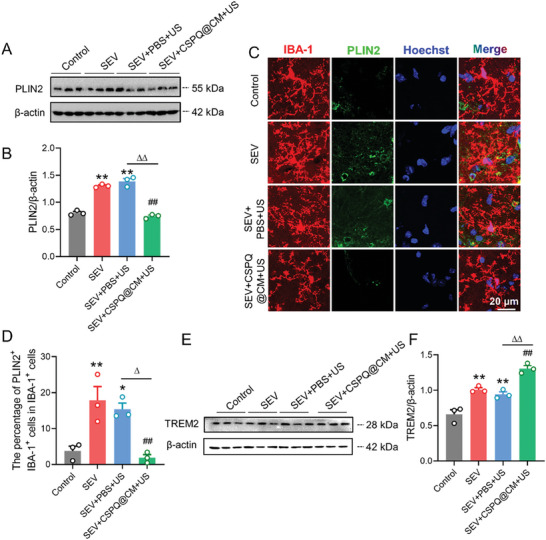
CSPQ@CM nanoparticles inhibited the lipid accumulation in the hippocampus of young mice induced by multiple sevoflurane exposures in their neonatal period. A,B) CSPQ@CM nanoparticles decreased the PLIN2 protein levels (*n* = 3 mice for each group). C) Immunostaining images of PLIN2 in microglia from the hippocampus (Scale bar = 20 µm). D) CSPQ@CM nanoparticles reduced the abundance of PLIN2 in the microglia from the hippocampus (*n* = 3 mice for each group). E,F) CSPQ@CM nanoparticles enhanced the TREM2 protein levels (*n* = 3 mice for each group). The two‐sided one‐way ANOVA with a Tukey post hoc analysis was used for comparison among multiple groups. Values were expressed as the mean ± SEM. *p < 0.05, **p < 0.01 versus Control group; ^##^ *p* < 0.01 versus SEV group; ^∆^ *p* < 0.05, ^∆∆^ *p* < 0.01 versus SEV+PBS+US group.

We also detected the PLIN2 protein level in the hippocampus of young mice on the PND 37. The lower PLIN2 protein levels were observed in the SEV+CSPQ@CM+US group of mice, in comparison with that of mice in the SEV+PBS+US group (**Figure** **10**A,B). The immunostaining was further carried out for characterization of PLIN2 and IBA‐1. Compared to the SEV+PBS+US group of mice, the amount of PLIN2 was significantly decreased in the microglia of hippocampus from the SEV+CSPQ@CM+US group mice (Figure 10C,D). Additionally, the microglial processes were also evaluated at the single cell level by morphometric analysis. The results show that multiple sevoflurane exposures significantly increased the fluorescence intensity of IBA‐1^+^ microglia and their soma size, and decreased their total process lengthen and the number of branches, which were partly reversed after treatment with CSPQ@CM nanoparticles (Figure S10A–E, Supporting Information). Finally, we determined the TREM2 protein level in the hippocampus of young mice on the PND37. The higher TREM2 protein levels were observed in the SEV+CSPQ@CM+US group of mice than that of mice in the SEV+PBS+US group (Figure 10E,F). These results further demonstrate that CSPQ@CM nanoparticles can alleviate microglial lipid accumulation and promote remyelination through TREM2 signaling in the developmental hippocampus of young mice induced by multiple sevoflurane exposures in their neonatal period.

## 3 Conclusion

Multiple sevoflurane exposures in the neonatal period can induce cognitive dysfunction in the young mice due to the abnormal lipid metabolism and pro‐inflammation of microglia. The in vitro results show that rationally designed CSPQ nanoparticles can obviously alleviate the lipid droplets accumulation by increasing the ApoE, ABCA1 and ABCG1 levels for promoting the efflux of cholesterol in BV2 cells. Simultaneously, they can also reduce the neuroinflammation by polarizing BV2 cells into M2‐like phenotype. Moreover, their protective effects against sevoflurane‐induced neurotoxicity can be abolished after the BV2 cells are transfected with siRNA‐TREM2. The knockdown of TREM2 can effectively increase lipid metabolism dysfunction in BV2 cells by blocking the increased ApoE, ABCA1 and ABCG1 levels caused by CSPQ nanoparticles. Moreover, knockdown of TREM2 also can promote the neuroinflammation by inhibiting microglial M2‐like polarization. More importantly, the in vivo results demonstrate that these nanoparticles can efficiently improve multiple sevoflurane exposures induced learning and memory deficits of mice by regulating TREM2 signaling pathway, decreasing microglial lipid accumulation, and promoting the remyelination.

In summary, this study has revealed the mechanism of CSPQ@CM nanoparticles attenuating the sevoflurane‐induced developmental neurotoxicity by improving microglial lipid metabolism and inhibiting the neuroinflammation for promoting remyelination via TREM2 signaling. It is of great significance for not only the treatment of developmental neurotoxicity induced by inhalational anesthetic agent, but also for the treatment of other neurodegenerative diseases such as Parkinson's disease and Alzheimer disease.

## 4 Experimental Section

### Materials

CuCl_2_·2H_2_O (> 99%), NaBH_4_ (> 99%), selenium powder (−100 mesh, ≥ 99.5%), fluorescence‐labeled amine‐modified polystyrene latex beads (1‐µm) and lipopolysaccharide (LPS) were bought from Sigma‐Aldrich (St. Louis, MO, USA). Murine IL‐4 was bought from PeproTech (Rocky Hill, NJ, USA). Polyvinylpyrrolidone powder was obtained from J&K Scientific Ltd (Beijing, China). Qe was provided by Adamas Reagent Ltd (Shanghai, China). 2,7‐dichlorofluorescein diacetate (DCFH‐DA) was obtained from Solarbio Technology Inc. (Beijing, China). The anti‐MBP and anti‐PLIN2 antibodies were purchased from Abcam (Cambridge, MA, USA). The anti‐iNOS antibody was acquired from Proteintech (Rosemont, IL, USA), anti‐Arg‐1 and anti‐TREM2 antibodies were purchased from Cell Signaling Technology, Inc. (Boston, MA, USA). The anti‐β actin mouse monoclonal antibody, HRP‐labeled goat anti‐rabbit/mouse IgG antibodies were obtained from Beyotime Biotechnology (Shanghai, China). Alexa Fluor 488‐labeled and cy3‐labeled secondary antibodies were purchased from Invitrogen (Waltham, MA, USA). Tissue‐Tek optimal cutting temperature (O.C.T) compound was provided by Sakura Finetek USA, Inc. (Torrance, CA, USA). The fixative solution for electron microscopy was obtained from Servicebio (Wuhan, China). The RIPA lysis buffer, poly‐D‐lysine, bicinchoninic acid (BCA) protein assay kit and Hoechst 33 342 were obtained from Beyotime Biotechnology (Shanghai, China). The enhanced chemiluminescence (ECL) kit and cell counting kit‐8 (CCK‐8) were purchased from New Cell & Molecular Biotech Co., Ltd (Shanghai, China). Polyvinylidene fluoride (PVDF) molecularly imprinted membrane was purchased from Millipore Ltd. (Shanghai, China). TRIzol reagent was acquired from Invitrogen (Waltham, MA, USA). The SuperMix for qPCR and universal SYBR qPCR Master were both supplied by Vazyme Biotech (Nanjing, China). BODIPY 493/503, fetal bovine serum (FBS) and penicillin‐streptomycin were purchased from Thermo Fisher Scientific (Waltham, MA, USA).

### Synthesis of Ultra‐Small Cu_2‐x_Se‐PVP (Abbreviated as CSP) Nanoparticles

Polyvinylpyrrolidone‐modified Cu_2‐_ *_x_* Se (abbreviated as CSP) nanoparticles were prepared according to the previously reported protocol.^[^ ^39^ ^]^ The resultant CSP nanoparticles were stored at 4 °C for further use.

### Preparation of Cu_2_‐_x_Se‐PVP‐Qe (Abbreviated as CSPQ) Nanoparticles

Cu_2‐_ *_x_* Se‐PVP‐Qe (CSPQ) nanoparticles were prepared according to previously described method.^[^ ^26^ ^]^ The resultant nanoparticles were referred as CSPQ nanoparticles.

### Synthesis of CSPQ@CM Nanoparticles

The CSPQ@CM nanoparticles were prepared according to the previous report.^[^ ^26^ ^]^ MES23.5 cells were cultured in a mixture of Dulbecco's Modified Eagle Medium/Ham's F‐12 (DMEM/F‐12, Gibco, Thermo Fisher) with 10% FBS and 1% antibiotics (penicillin/streptomycin). Then, they were collected and suspended by an ice‐cold Tris buffer, which contained 10 mm MgCl_2_, 10 mm Tris and 1×ethylenediamine tetra‐acetic acid free protease inhibitor for 1 h at 4 °C (pH = 7.4). The obtained suspension was sonicated for 10 min in an Ultrasonic Cell Disruption System with an ice‐bath, followed by centrifugation at 11 480 rpm for another 10 min, and centrifuged at 110 000 rpm for 1 h (Optima L‐100XP, Beckman Coulter, Inc.) to obtain the cell membrane. The cell membrane was re‐dispersed with CSPQ nanoparticles solution and extruded through 400 nm polycarbonate membranes for five cycles to get the CSPQ@CM nanoparticles.

### Animals

Eighteen female and six male healthy C57BL/6J mice (weighing 20–25 g) in breeding period were obtained from the Experimental Animal Centre of Soochow University (Animal license No. SYXK Jiang‐su 2017‐0043) to generate the off‐spring mice. The male neonatal mice were sequentially numbered with ear tags and randomly divided into different groups using the online randomization tool (https://www.randomizer.org/). All animals were fed with standard diets under the controlled environment with 12 h light/dark cycle, 40% – 60% relative humidity and room temperature of 24 ˚C – 26 ˚C. Animal experimental protocol was approved by the Ethics Committee for Animal Experimentation of Soochow University. All experimental procedures complied with the Guide for the Care and Use of Laboratory Animals published by the US National Institutes of Health (*NIH Publication No. 85‐23, revised in 1996*).

### Multiple Sevoflurane Exposures

As previously described,^[^ ^3^, ^37^ ^]^ the neonatal mice were received 3% sevoflurane and 60% oxygen (balanced with nitrogen) for 2 h (2 L min^−1^ fresh gas for 3 min, followed by 1 L min^−1^) in a chamber for three days using the Datex‐Ohmeda anesthesia system (Madison, WI, USA) on the postnatal day (PND) 6, 8, and 10, respectively. The sevoflurane concentration was monitored and adjusted by using a gas analyzer (Vamos; Dräger Medical, Germany). The mice from the Control group received 60% oxygen in nitrogen for 2 h. The rectal temperature of mice was maintained at 37 ± 0.5 °C. After treatment, the mice were returned to home cages under standard care.

### Injection of CSPQ@CM Nanoparticles

To detect the protective effect of CSPQ@CM nanoparticles on sevoflurane‐induced developmental neurotoxicity, the mice were randomly divided into five groups: (I) normal healthy mice (Control group); (II) normal healthy mice intravenously injected with phosphate buffered saline (PBS) after ultrasound treatment (Control +PBS+US group); (III) the mice received multiple sevoflurane exposures (SEV group); (IV) the mice in sevoflurane group injected with PBS followed by sonication with focused ultrasound (SEV+PBS+US group); (V) the mice in sevoflurane group injected with CSPQ@CM nanoparticles followed by sonication with focused ultrasound (SEV+CSPQ@CM+US group). To improve the accumulation of nanoparticles in the brain, the US transducer (1 MHz and 37 mm diameter) was used to open the BBB of mice temporarily, which was driven by a function generator connected to a power amplifier. A movable cone filled with degassed water was employed to hold the transducer in place and direct the US beam into the brain. The sonication parameters were set at 0.6 MPa acoustic pressure, 0.5 MHz frequency, 1 ms pulse interval, and 90 s sonication time as the previously described.^[^ ^26^ ^]^ Before sonication, 50 µL of microbubbles with a mean diameter of 2 µm and concentration of 1 × 10^9^ bubbles mL^−1^ were intravenously injected into mice via the tail vein. After sonication, CSPQ@CM nanoparticles were intravenously injected twice at a dose of 12 mg kg^−1^ on PND 27 and 29, respectively. Besides, Evans blue (2% wt) was used to examine the opening of the blood brain barrier (BBB), and the staining of brain tissues with rubeanic acid (RA) was performed for confirm the deposition of nanoparticles in the brain.^[^ ^25^ ^]^

### Behavioral Tests

To investigate the long‐term effect of multiple sevoflurane exposures in neonatal period on the cognitive function of young mice, we carried out the Morris water maze (MWM) test, the open field test, and the novel‐object recognition (NOR) test. The behavioral tests were performed with two cohorts of mice as previously described.^[^ ^40^ ^]^ The MWM tests were performed with the first cohort mice on the PND 31–36. The open field and NOR tests were performed with the second cohort mice on the PND 35‐36. About 24 h after the open field test, the mice were allowed to start the NOR tests. The investigators were blinded to group allocation during data collection and analysis. In all behavioral tests, the experimental groups were kept blinded to the investigators, and data collection and analysis were performed by different investigators.

### MWM Test

The MWM test was performed to detect the cognitive impairment in mice.^[^ ^3^ ^]^ Briefly, water maze device was filled with opaque water to reach the level of 1.0 cm above the surface of a platform (10 cm in diameter). During the experiment, the water temperature was kept at 22°C and the surrounding environment remained quiet. In the training phase on the PND 31–35, the mice were trained to reach the platform for 5 days with 4 trials per day, and the escape latency (time for mice to reach the platform) was recorded to evaluate their spatial learning. In the testing phase on the PND 36, the platform was removed. The mean distance spent in the target quadrant, the time spent in the target quadrant, the frequency of crossing the platform and the swimming speed of mice were recorded for assessing their memory function. A heat lamp was used to warm and dry the mice before they returned to home cages. SuperMaze Morris video analysis system (XR‐XM101, Shanghai Xinruan Information Technology Co., Ltd, Shanghai, China) was used to record and analyzed.

### Open Field Test

The open field test was carried out to evaluate locomotor activity in mice.^[^ ^41^ ^]^ The case size for the open field test was 50 cm × 50 cm × 50 cm (length × width × height). The mice on the PND 35 were placed in the assigned position, and the SuperMaze video analysis system (XR‐XZ301, Shanghai Xinruan Information Technology Co., Ltd) was used to record their activity for the next 10 min. The mean speed, time spent in central and peripheral regional, and number of regional transitional of each mouse were recorded and analyzed. At the end of the test, the mice were returned to their home cages, and the apparatus was cleaned with 70% ethanol.

### NOR Test

The NOR test has been developed to study learning and memory in rodents. It is based on the principle that mice have instinct to explore new objects.^[^ ^42^ ^]^ The mice were first individually placed in the empty open field (50 cm × 50 cm × 50 cm) for 10 min to adapt to the environment on the PND 36. The familiarization trail was performed at 24 h after the habituation session. Two identical cubes (8 cm side length) were placed in the open field, 5 cm away from the wall. The mice were individually placed in the apparatus and allowed to freely explore for 10 min. After an interval of 1 h, the test session was performed. The mice were then placed back into the chamber with one new object and one familiar object. The cylinder acted as the new object in the present experiment. Then, the mice were individually allowed to explore for 10 min. The exploration time of the familiar object and the novel object was recorded for analysis (XR‐XZ301, Shanghai Xinruan Information Technology Co., Ltd). The discrimination index was calculated as follows: Discrimination index = (% time with novel object − % time with familiar object) / (% time with novel object + % time with familiar object).

### Transmission Electron Microscopy and G‐Ratio Quantification

After the mice were deeply anaesthetized and perfused transcardially with PBS on the PND 37, their hippocampus was dissected immediately from the brain. The tissues of CA1 area were made from 1 mm × 1 mm × 1 mm small cubes, which were immersed in the electron microscopy fixative solution for 4 h. Then, the cubes were fixed in osmic acid in 0.1 mol L^−1^ PBS (pH7.4), followed by dehydration in ethanol and infiltration of Spurr's resin. The tissues were embedded in the Spurr's resin. After the polymerization of resin, 70 nm slices were cut and stained with uranyl acetate and lead citrate. Images were captured by a Transmission Electron Microscope (Hitachi HT7700, Tokyo, Japan). Remyelination was analyzed by counting the number of naked axons and the number of myelinated axons per field, with the minimum of ten fields being analyzed. The G‐ratio was quantified by dividing the axonal diameter by the myelinated fiber diameter.

### The Culture of Microglial BV2 Cells

The immortalized microglial BV2 cell line has been used extensively in the research associated with neurodegenerative disorders.^[^ ^43^ ^]^ The BV2 cells were cultured in DMEM with 10% FBS and penicillin‐streptomycin (100 µg mL^−1^) in an incubator (Thermo Fisher Scientific, Waltham, MA, USA) at 37 °C with 5% CO_2_. They were incubated with 80 µm CSPQ nanoparticles for 12 h, followed by culture with fresh medium containing 1 mm sevoflurane for another 6 h.

### The Cell Viability Assay

The cell viability was measured by the cell counting kit (CCK)−8 according to the previous study.^[^ ^44^ ^]^ Briefly, BV2 cells were seeded into 96‐well plates at a density of 1 ×10^4^ cells per well for overnight, and incubated with different concentrations (0, 20, 40, 80, 100 µm) of CSPQ nanoparticles for 12 h, respectively. After incubation with 10 µL CCK‐8 solution for 2 h, a multimode plate reader (PerkinElmer, Waltham, MA, USA) was used to measure the absorbance at 450 nm.

### The Intracellular ROS Determination

The generation of ROS was detected by 2,7‐dichlorofluorescein diacetate (DCFH‐DA) probe.^[^ ^26^ ^]^ When the DCFH‐DA penetrated into cells, it changed into non‐fluorescent DCFH through the dissociation of the acetyl group. The freshly formed DCFH was rapidly oxidized by the intracellular ROS into DCF with bright fluorescence, which was proportional to the amount of ROS.

Briefly, BV2 cells were seeded onto 35 mm glass‐bottom dishes and cultured for 24 h, and then pre‐treated with CSPQ nanoparticles (80 µm) for 12 h. After that, the cells were cultured for another 6 h with medium containing 1 mm sevoflurane. Next, the cells were washed with PBS for twice, and followed by staining with 10 µm DCFH‐DA for 30 min and the nuclei were immersed with Hoechst 33 342 (1:1000) for 10 min at 37 °C. The fluorescence images were recorded with a confocal laser scanning microscope (CLSM, FV1200, Olympus, Japan).

### BODIPY Staining

BODIPY staining was carried out to detect the accumulation of lipid droplets in the microglia as the previously described.^[^ ^35^ ^]^ Microglial BV2 cells were seeded on poly‐L‐lysine coated glass slides with a density of 4 ×10^4^ cells per slide and cultured for 12 h. After pretreated with 80 µm CSPQ nanoparticles for 12 h, the BV2 cells were further cultured with 1 mm sevoflurane for another 6 h. Subsequently, the cells were fixed with 4% paraformaldehyde for 30 min, washed three times with PBS and incubated with BODIPY 493/503 (1:1000 from 1 mg mL^−1^ stock solution in DMSO) and Hoechst 33 342 (1:1000) for 10 min at room temperature. Finally, the BV2 cells were washed twice by PBS solution, and observed with CLSM (FV1200, Olympus, Japan).

### Microglial Phagocytosis Assay

Phagocytosis assay was performed as previously described.^[^ ^45^ ^]^ Microglial BV2 cells were seeded onto 35 mm glass‐bottom dishes at a density of 4 ×10^4^ cells per well. After treatment, the red fluorescence labeled amine‐modified polystyrene latex beads (1‐µm) were used to assay phagocytosis. Fluorescence labeled latex beads were pre‐opsonized in FBS at the ratio of 1: 2 (v: v) for 1 h at 37 °C, and the solution were further mixed with DMEM containing 10% FBS at the ratio of 1: 1000 (v: v). After incubating with the pretreated latex beads for 2 h at 37 °C, the BV2 cells were washed with PBS for three times to remove the non‐phagocytized beads. Next, the cells were fixed by 4% paraformaldehyde for 30 min at room temperature, and stained with Hoechst 33 342 (1:1000) for 10 min. Finally, the phagocytosis of BV2 cells was observed under with CLSM (FV1200, Olympus, Japan). For the quantification, at least 10 different fields were randomly selected from each well and counted the positive staining signal using Image J 6.0 software (National Institutes of Health, Bethesda, Maryland, USA). The phagocytosis capability was determined by calculating the relative fluorescence intensity of latex beads and the ratio of cells labeled with fluorescence to the total BV2 cells.

### Immunofluorescence Staining

Microglial BV2 cells were seeded onto 35 mm glass‐bottom dishes with a density of 4 ×10^4^ cells per dish. After the cells adhered to the wall, they were pretreated with 80 µm CSPQ nanoparticles for 12 h and stimulated with 1 mm sevoflurane for another 6 h. The cells were washed with PBS twice and fixed with 4% paraformaldehyde for 30 min. Then, they were incubated with 1% Triton for 10 min and washed with PBS for three times (5 min for each time). After the cells were blocked with 5% bovine serum at 37 °C for 2 h, they were incubated with primary antibodies for overnight in the dark at 4 °C. The primary antibodies included anti‐IBA‐1 (1:400, Abcam), anti‐iNOS (1:50, Proteintech) and anti‐Arg‐1 (1:50, Cell Signaling Technology).

Besides, after the mice were intracardially perfused with PBS followed by 4% paraformaldehyde on the PND 37, their brain tissues were removed for immersion in 4% paraformaldehyde, dehydrated with sucrose solution in PBS, and then embedded in optimal cutting temperature (O.C.T.) compound. Brain slices with a thickness of 20 µm were obtained by using a freezing microtome (Leica CM 1950, Wetzlar, Germany). After blocked in PBS with 0.1% Triton and bovine serum, the slices were stained with primary antibodies, including anti‐IBA‐1 (1:400, Abcam), anti‐MBP (1:200, Abcam) and anti‐PLIN2 (1:200, Abcam). Then, the cells or brain slices were incubated with Alexa Fluor 488‐labeled and cy3‐labeled secondary antibodies for 1 h in the dark at 37 °C. Finally, the cells or brain slices were stained with Hoechst 33 342 (1:1000) for 10 min and the fluorescence images were monitored by CLSM (FV1200, Olympus, Japan). To quantify the intensity of immunoreactivity, three independent microscopic fields in each brain section were randomly acquired in the hippocampal CA1 region. Three sections per mice were imaged, and a mean count per mouse was used for statistical evaluation. The analysis of cell counts and colocalization were conducted using ImageJ software (Fiji edition, National Institutes of Health, Bethesda, Maryland, USA) by an observer blind to condition.

For the analysis of microglial morphology, Z‐stack images were acquired at 0.5‐µm intervals in a 15‐µm Z‐range using Olympus FV1200 confocal microscope and Z‐stack images were converted into single‐plane maximal intensity projection. Microglia were divided into ramified morphology and amoeboid morphology. The cells with smaller cell bodies and long thin branches were classified as ramified morphology, and those rounded in shape with enlarged cell bodies were defined as amoeboid morphology.^[^ ^46^ ^]^ The morphological analysis, such as soma size, total process length and number of branches for individual cells was carried out to evaluate the changes of microglial morphology.

### Western Blot

Similar to the previous report,^[^ ^47^ ^]^ the total protein in BV2 cells or hippocampus was extracted by using the RIPA reagents, and the protein concentration was determined with a bicinchoninic acid (BCA) protein assay kit. Then, the protein was separated by the sodium dodecyl sulfate‐polyacrylamide gel electrophoresis (SDS‐PAGE). After the cell or tissue proteins in the SDS‐PAGE gels were transferred onto polyvinylidene fluoride (PVDF) membranes, they were blocked in 5% milk solution for 2 h at room temperature. Then, the PVDF membranes were incubated with the primary antibodies overnight at 4 °C, and the protein levels were normalized by using β‐actin as a control. After washed by TBST solution for three times, the PVDF membrane was incubated with the horseradish peroxidase (HRP)‐conjugated secondary antibodies for 2 h at room temperature. Finally, the protein bands were detected using an electrochemiluminescence (ECL) kit with an imaging system (FluorChem M, Alpha Inotech Corp., San Leandro, CA, USA). Image J 6.0 software (National Institutes of Health, Bethesda, Maryland, USA) was used for quantitative analysis. The following primary antibodies were used: anti‐TREM2 (1:1000, Abcam), anti‐MBP (1:1000, Abcam), anti‐PLIN2 (1:1000, Abcam) and anti‐β‐actin (1:1000, Beyotime Biotechnology).

### Quantitative Real‐Time PCR

The total RNA from the BV2 cells was extracted by using the previously reported TRIzol reagent,^[^ ^47^ ^]^ and the absorbances at 260 and 280 nm were measured for RNA quantification. According to the manufacturer's instructions, the reverse transcription was performed using the cDNA Synthesis Kit in 20 µL volume. Quantitative PCR was conducted with the SYBR qPCR Master in 15 µL volume on Roche Light Cycler R480 System (Roche, Bedford, MA, USA). The reaction mixture contained 5 µL of cDNA, 0.4 µL of each pair of primers, 7.5 µL SYBR Green and 1.7 µL ddH_2_O water. The cycle parameters were set as follows: pre‐denaturation at 95 °C for 10 s, denaturation at 60°C for 15 s, and annealing at 72 °C for 20 s for 40 cycles. The value obtained for the target gene expression was normalized to GAPDH and analyzed by the relative gene expression 2^−ΔΔCT^ method. All the experiments were repeated three times. The primers were provided by Shanghai Sangon Co., Ltd. Primer sequences were listed in **Table** **1**.

**Table 1:** Primers for quantitative real‐time PCR.

Gene	Forward primer	Reverse primer
IL‐1β	GAAATGCCACCTTTTGACAGTG	TGGATGCTCTCATCAGGACAG
IL‐6	ATAGTCCTTCCTACCCCAATTTCC	GATGAATTGGATGGTCTTGGTCC
TGF‐β	CACTGATACGCCTGAGTG	GTGAGCGCTGAATCGAAA
IL‐10	TAAAAGCAAGGCAGTGGAGC	GATGCCGGGTGGTTCAATTT
iNOS	GTTCTCAGCCCAACAATACAAGA	GTGGACGGGTCGATGTCAC
Arg‐1	CTCCAAGCCAAAGTCCTTAGAG	GGAGCTGTCATTAGGGACATCA
TREM2	CTGGAACCGTCACCATCACTC	CGAAACTCGATGACTCCTCGG
ABCA1	GACCATGAAAGTGACACGCTG	CCTCTTCCACAAAAGGGCCA
ABCG1	GGCCATGGTGTGTGGTTCTA	CGCCCCAATGCTACCTAGAG
ACAT1	TTTGCTGATGCTGCCGTAGA	ATCCCAATTGGATGGCCCAG
ACAT2	TGGGAGTCGCAATGTGTGTT	TGCTGCCCACCTTTCAACAA
APOE	CTCCCAAGTCACACAAGAACTG	CCAGCTCCTTTTTGTAAGCCTTT
GAPDH	GGTTGTCTCCTGCGACTTCA	TGGTCCAGGGTTTCTTACTCC

### The Intracellular Cholesterol Content Analysis

After BV2 cells were separated by centrifugation at 1000 rpm for 10 min, the supernatant was discarded, and the cells were washed with PBS for twice. Then, BV2 cells were resuspended in 300 µL PBS, and sonicated in an ice bath (300 W power, 3–5 seconds/time, 30 seconds interval, repeated 3–5 times). After that, 2.5 µL Milli‐Q H_2_O, calibration solution, sample solution with 250 µL working solution were added into 96 well plates and incubated for 10 min, respectively. The absorbance of formazan at 510 nm was measured by a microplate reader (PerkinElmer EnSpire, Singapore). The relative intracellular cholesterol content was calculated by the following formula. Intracellular cholesterol content (mmol/g protein)  = [OD (sample) − OD (blank)] / [OD (calibrate) − OD (blank)] × the content of calibrate (mmol/L)/ the protein content of sample (g protein/L)

### TREM2‐siRNA Transfection

The small interfering RNA (siRNA) targeted TREM2 gene (NM_03 1254) was produced by GenePharma (Shanghai, China). For knockdown of TREM2, siRNA‐TREM2 transfection was performed in BV2 cells as previously described.^[^ ^48^ ^]^ BV2 cells were randomly divided into five groups: Control group, negative siRNA group, siRNA1 group, siRNA2 group and siRNA3 group. Briefly, the cells were seeded in six‐well plates with a density of 4 × 10^5^ cells per well and cultured at 37 °C for 24 h. According to the manufacturer's protocol, the TREM2 siRNAs or negative siRNA were gently mixed with Opti‐MEM medium, respectively. Then, the siRNA solution was mixed with the Lipofectamine 3000 (Invitrogen; Thermo Fisher Scientific, Inc., Waltham, MA, USA) and further incubated for 15 min at room temperature. After that, BV2 cells were washed with PBS for three times and incubated with siRNA transfection solution (with the terminate concentration of siRNA‐TREM2 at 75 nm) in a CO_2_ incubator for 5 h at 37 °C. The medium was aspirated and replaced with fresh normal growth medium. After transfection for 24 h or 48 h, Western blotting was performed to assess transfection efficiency. Besides, the cell mortality was calculated to ensure the reliability of the transfection data. Under the transfection condition, the cell mortality was less than 10%, which could satisfy the subsequent experiments. The interference efficiency of siRNA‐TREM2 was confirmed by the Western blotting, and siRNA oligo sequences were shown in **Table** **2**.

**Table 2:** RNA oligo for TREM2‐siRNA transfection

Gene	Sense	Antisense
siRNA1	CCCUCUAGAUGACCAAGAUTT	AUCUUGGUCAUCUAGAGGGTT
siRNA2	CCUCCUCUUUCCAAGGAAUTT	AUUCCUUGGAAAGAGGAGGTT
siRAN3	UCUCCUGAGCAAGUUUCUUTT	AAGAAACUUGCUCAGGAGATT
Negative	UUCUCCGAACGUGUCACGUTT	ACGUGACACGUUCGGAGAATT

### Statistical Analysis

All data were expressed as mean ± standard error of the mean (SEM) and analyzed by the GraphPad Prism software (version 8.0, GraphPad, San Diego, CA, USA). Statistical significance was determined by using Student's *t*‐test, one‐way analysis of variance (ANOVA) or two‐way ANOVA. *p* < 0.05 was considered statistically significant.

## Conflict of Interest

The authors declare no conflict of interest.

## Supporting information

Supporting Information

## Data Availability

The data that support the findings of this study are available from the corresponding author upon reasonable request.
