# Supplementary material for: Boosting Microglial Lipid Metabolism via TREM2 Signaling by Biomimetic Nanoparticles to Attenuate the Sevoflurane‐Induced Developmental Neurotoxicity
Source: Adv Sci (Weinh). 2023 Dec 25;11(10):2305989. doi: 10.1002/advs.202305989 (PMC10933683; doi:10.1002/advs.202305989)
Supplement: Supplementary file 1 — Supporting Information [file ADVS-11-2305989-s001.pdf]

ADVANCED SCIENCE
Open Access

Supporting Information

for *Adv. Sci.*, DOI 10.1002/advs.202305989

Boosting Microglial Lipid Metabolism via TREM2 Signaling by Biomimetic Nanoparticles to Attenuate the Sevoflurane-Induced Developmental Neurotoxicity

Wenting Li, Xiaowen Meng, Ke Peng, Yaobao Han, Hanghang Liu, Weiming Zhao, Gang Wang, Li Deng, Hong Liu, Zhen Li and Fuhai Ji**

Supporting information

Boosting Microglial Lipid Metabolism *via* TREM2 Signaling by Biomimetic Nanoparticles to Attenuate the Sevoflurane-induced Developmental Neurotoxicity

Wenting Li[1,2,3], Xiaowen Meng[1,3], Ke Peng[1,3], Yaobao Han[2], Hanghang Liu[2], Weiming Zhao[1,3], Gang Wang[1, 2, 3], Li Deng[1,3], Hong Liu[4], Zhen Li[2,*], Fuhai Ji[1, 3, *]

[1]Department of Anesthesiology, the First Affiliated Hospital of Soochow University, Suzhou, Jiangsu, 215006, China

[2]Center for Molecular Imaging and Nuclear Medicine, State Key Laboratory of Radiation Medicine and Protection, School for Radiological and Interdisciplinary Sciences (RAD-X), Suzhou Medical College, Soochow University, Collaborative Innovation Center of Radiation Medicine of Jiangsu Higher Education Institutions, Suzhou, 215123, China

[3]Institute of Anesthesiology, Soochow University, Suzhou, Jiangsu, 215006, China

[4]Department of Anaesthesiology and Pain Medicine, University of California Davis Health, Sacramento, CA 95817, USA

* Corresponding authors:

Zhen Li, E-mail address: zhenli@suda.edu.cn

Fuhai Ji, E-mail address: jifuhai@suda.edu.cn

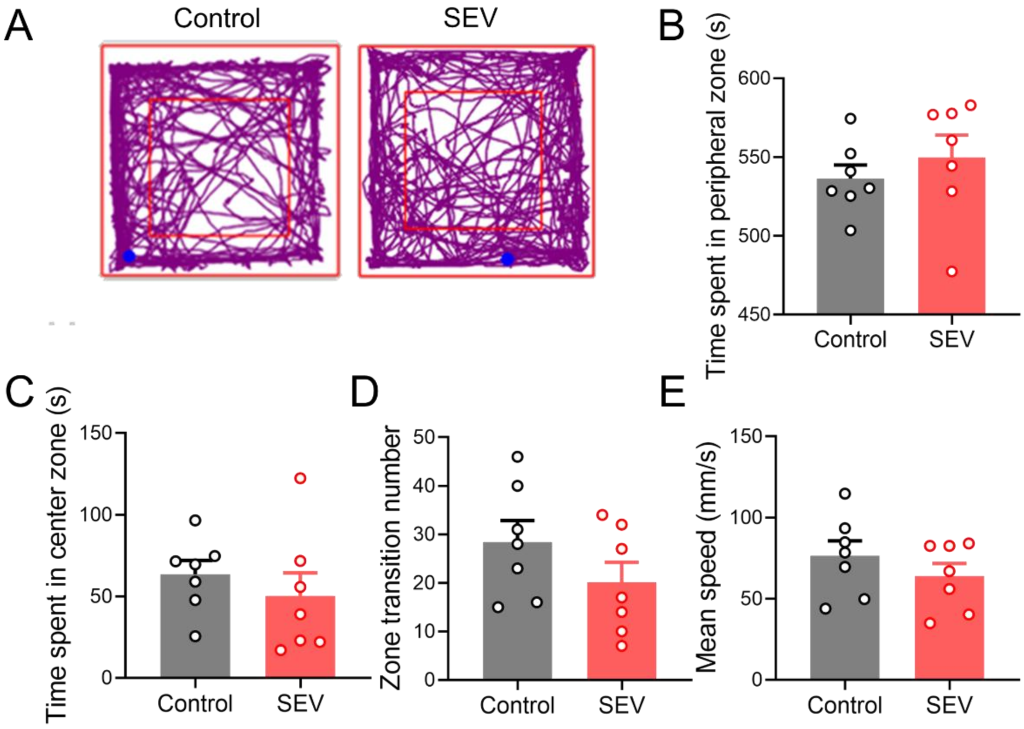

Figure S1. The effect of multiple sevoflurane exposures on the spontaneous locomotor activity of mice. (A) Representative motion track of mice obtained from the open field test. (B) The time of mice spending in the peripheral zone (n = 7 mice for each group). (C) Time of mice spending in the center zone (n = 7 mice for each group). (D) The number of zone transition (n = 7 mice for each group). (E) The mean speed of mice (n = 7 mice for each group). The two-sided unpaired Student's t-test was used for comparison between two groups. Values were expressed as the mean ± SEM.

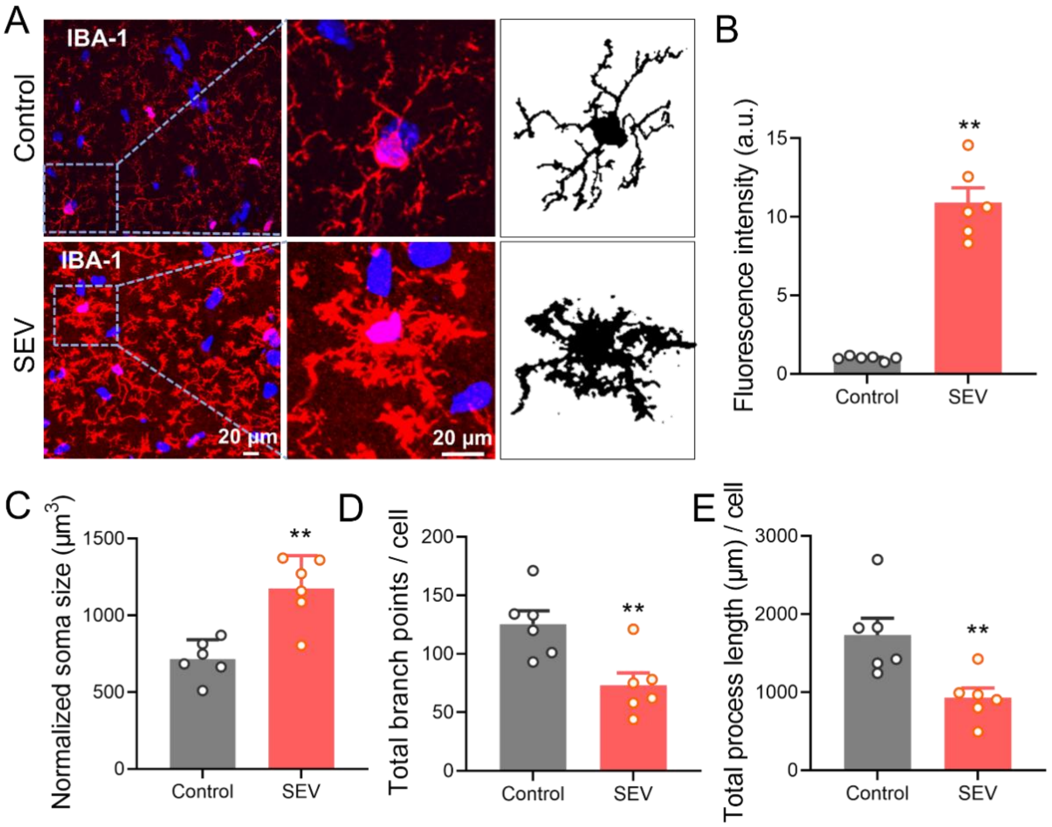

Figure S2. Multiple sevoflurane exposures activated microglia in the hippocampus of mice. (A) Multiple sevoflurane exposures induced more amoeboid morphology of microglia in the hippocampus (Scale bar = 20 μm). (B) The fluorescence intensity of IBA-1^+ microglia (n = 6 mice for each group). (C) The normalized soma size (n = 6 mice for each group). (D) The number of branch points of IBA-1^+ microglia (n = 6 mice for each group). (E) The total process length (n = 6 mice for each group). The two-sided unpaired Student's t-test was used for comparison between two groups. Values were expressed as the mean ± SEM. *$p < 0.05$, **$p < 0.01$ *vs* Control group.

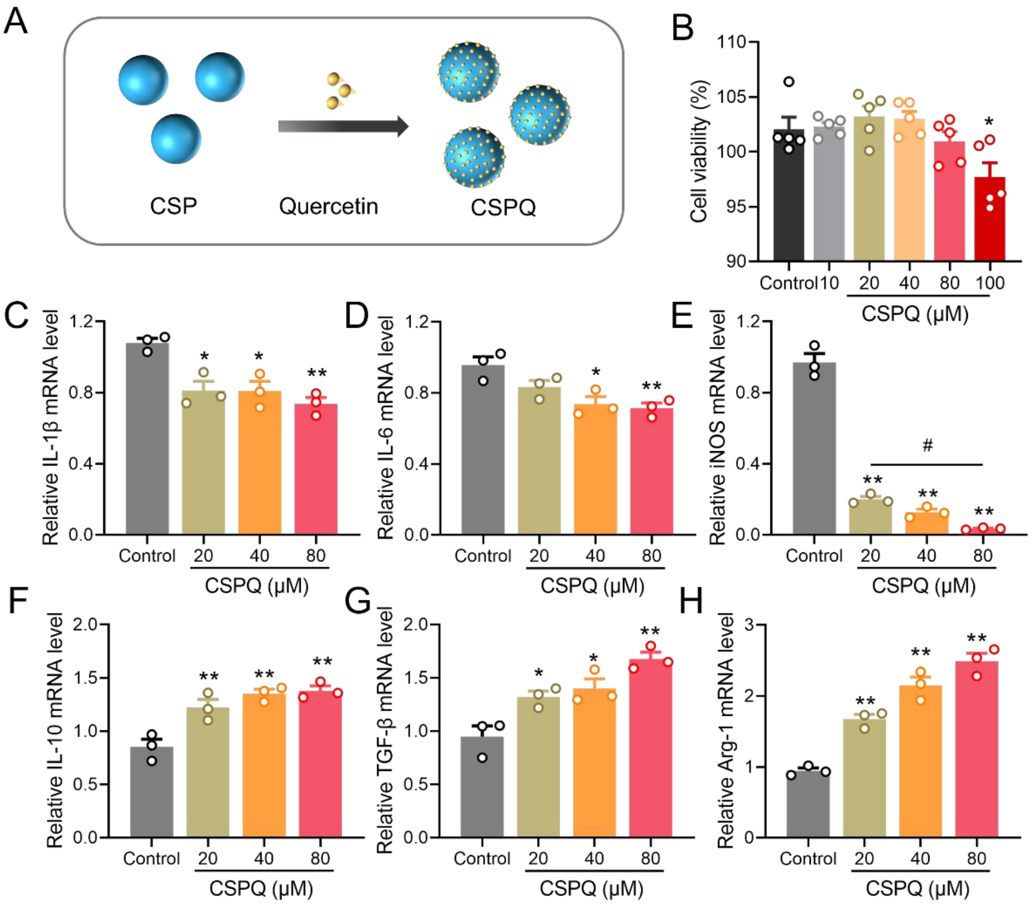

Figure S3. CSPQ nanoparticles promoted the polarization of microglia into M2-like phenotype. (A) Schematic illustration of the preparation of CSPQ nanoparticles. (B) The cytotoxicity of CSPQ nanoparticles towards BV2 cells (n = 5 wells for each group). (C, D) Proinflammatory cytokine IL-1β and IL-6 mRNA levels (n = 3 wells for each group). (E) M1 phenotype microglia marker iNOS mRNA levels (n = 3 wells for each group). (F, G) Anti-inflammatory cytokine IL-10 and TGF-β mRNA levels (n = 3 wells for each group). (H) M2 phenotype microglia marker Arg-1 mRNA levels (n = 3 wells for each group). The two-sided one-way ANOVA with a Tukey post hoc analysis was used for comparison among multiple groups. Values were expressed as the mean ± SEM. *$p < 0.05$, **$p < 0.01$ *vs* Control group; $^{\#}p < 0.05$ *vs* 20 μM CSPQ group.

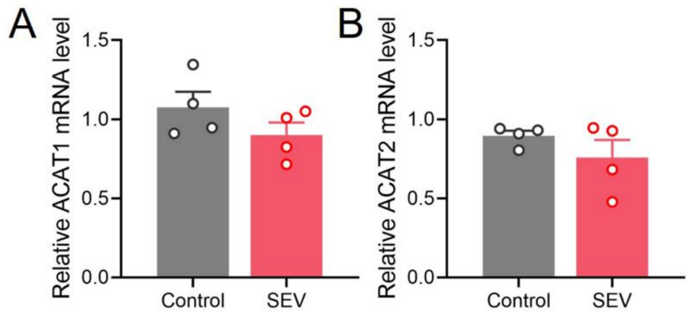

Figure S4. Multiple sevoflurane exposures did not alter the mRNA levels of ACAT1 and ACAT2 in BV2 cells. (A) ACAT1 mRNA levels (n = 4 wells for each group). (B) ACAT2 mRNA levels (n = 4 wells for each group). The two-sided unpaired Student's t-test was used for comparison between two groups.

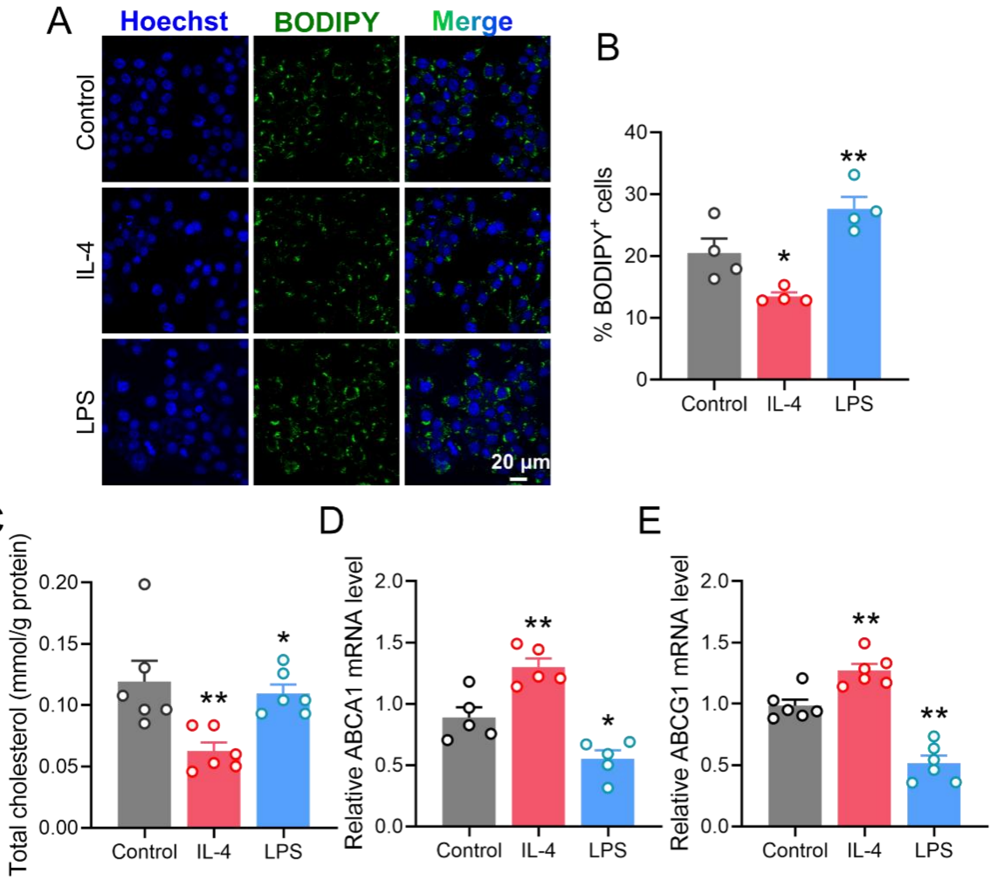

Figure S5. Microglial M2-like polarization facilitated the lipid metabolism in BV2 cells. (A) Immunofluorescence images of BV2 cells for detecting the intracellular accumulation of lipid droplets (blue: cell nuclei stained by Hoechst 33342 dye; green: BODIPY, scale bar = 20 μm). (B) IL-4 decreased the accumulation of lipid droplets, but LPS increased the accumulation of lipid droplets in BV2 cells (n = 4 wells for each group). (C) IL-4 decreased the intracellular cholesterol content, but LPS increased the intracellular cholesterol content in BV2 cells (n = 6 wells for each group). (D, E) IL-4 increased the ABCA1 and ABCG1 mRNA levels, but LPS decreased the ABCA1 and ABCG1 mRNA levels in BV2 cells (n = 5 or 6 wells for each group). The two-sided one-way ANOVA with a Tukey post hoc analysis was used for comparison among multiple groups. Values were expressed as the mean ± SEM. *$p < 0.05$, **$p < 0.01$ *vs* Control group.

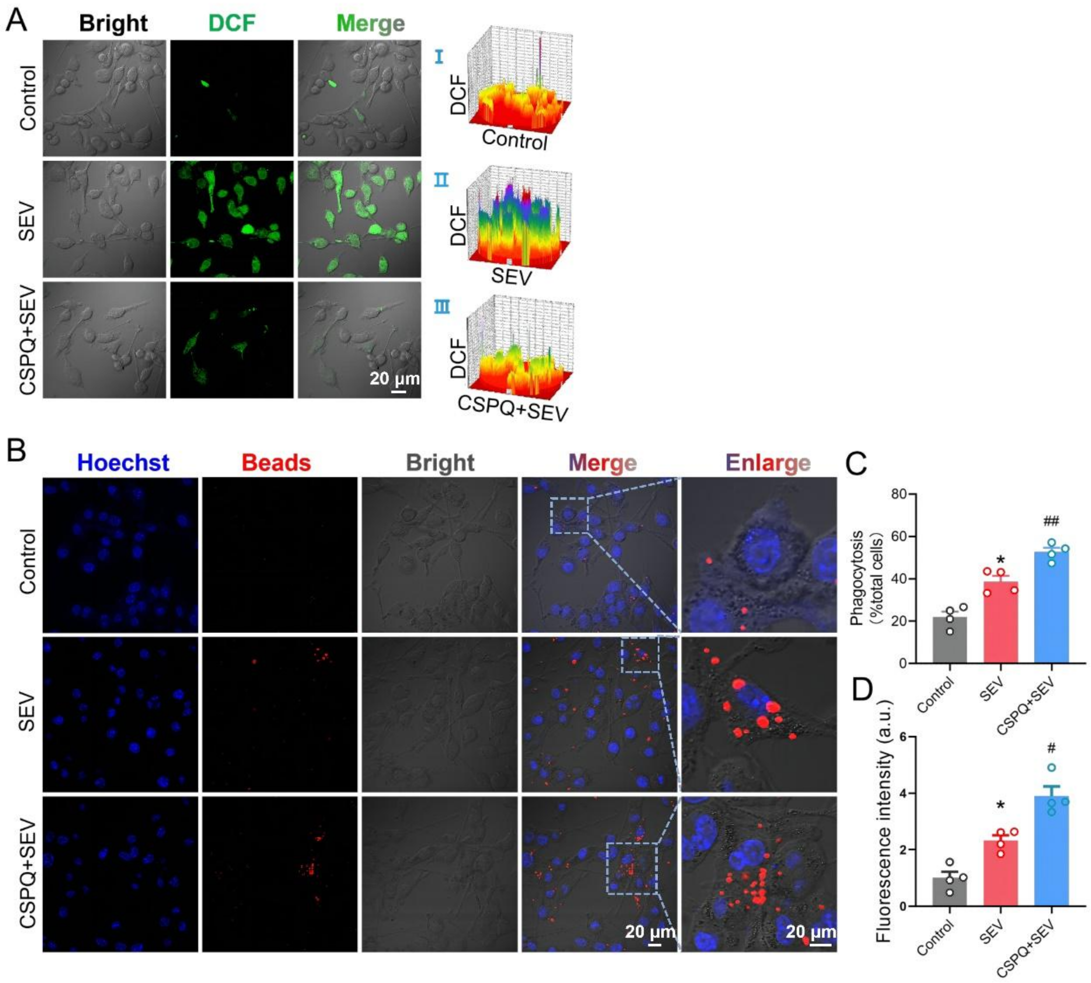

Figure S6. CSPQ nanoparticles protected microglia by scavenging the ROS and enhancing the phagocytosis capability. (A) Immunofluorescence images of BV2 cells for detecting the intracellular ROS, and CSPQ nanoparticles inhibited the production of intracellular ROS induced by sevoflurane (blue: cell nuclei stained by Hoechst 33342 dye; green: DCF fluorescence, scale bar = 20 μm). The fluorescence distribution and intensity in BV2 cells were shown in I-III. (B) Immunofluorescence images of BV2 cells for determining their phagocytosis capability, and the effect of CSPQ nanoparticles on phagocytosis capability (blue: cell nuclei stained by Hoechst 33342 dye; red: the microbeads). (C, D) The phagocytosis capability was expressed as the relative fluorescence of latex beads and the ratio of cells labeled with fluorescent latex beads to the total BV2 cells (n = 4 wells for each group). The two-sided one-way ANOVA with a Tukey post hoc analysis was used for comparison among multiple groups. Values were expressed as the mean ± SEM. *$p < 0.05$, $^{\#}p < 0.05$, $^{\#\#}p < 0.01$ *vs* Control group.

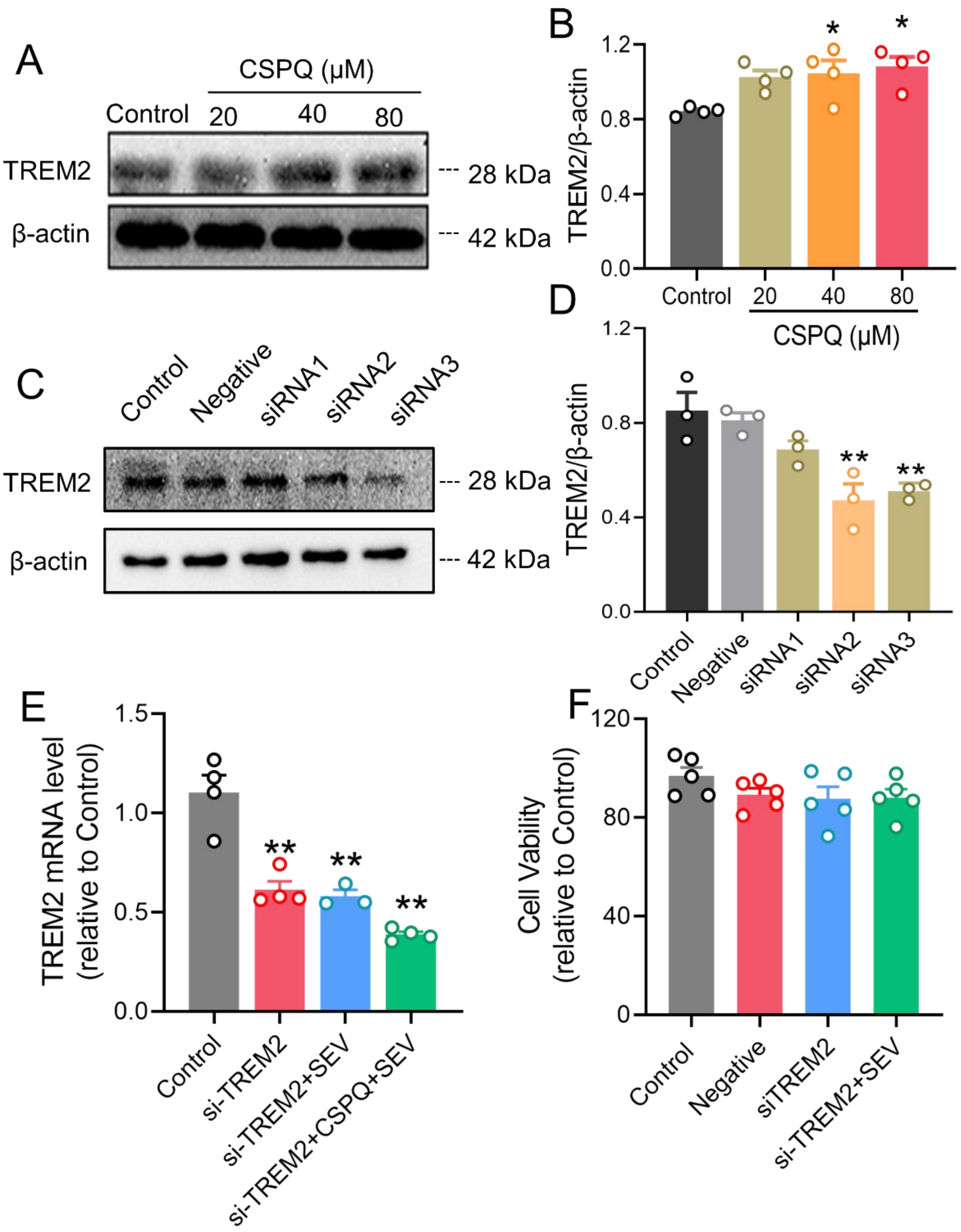

Figure S7. siRNA-TREM2 transfection obviously inhibited the increased mRNA expression of TREM2 in BV2 cells. (A, B) CSPQ nanoparticles increased the TREM2 protein level (n = 4 for each group). (C, D) Transfection with siRNA-TREM2 reduced TREM2 protein expression in BV2 cells (n = 3 wells for each group). (E) siRNA-TREM2 transfection inhibited the increased mRNA of TREM2 induced by sevoflurane or CSPQ nanoparticles (n = 4 wells for each group). (F) The cytotoxicity of siRNA-TREM2 towards BV2 cells (n = 5 wells for each group). The two-sided one-way ANOVA with a Tukey post hoc analysis was used for comparison among multiple groups. Values were expressed as the mean ± SEM. $^{**}p < 0.01$ *vs* Control group.

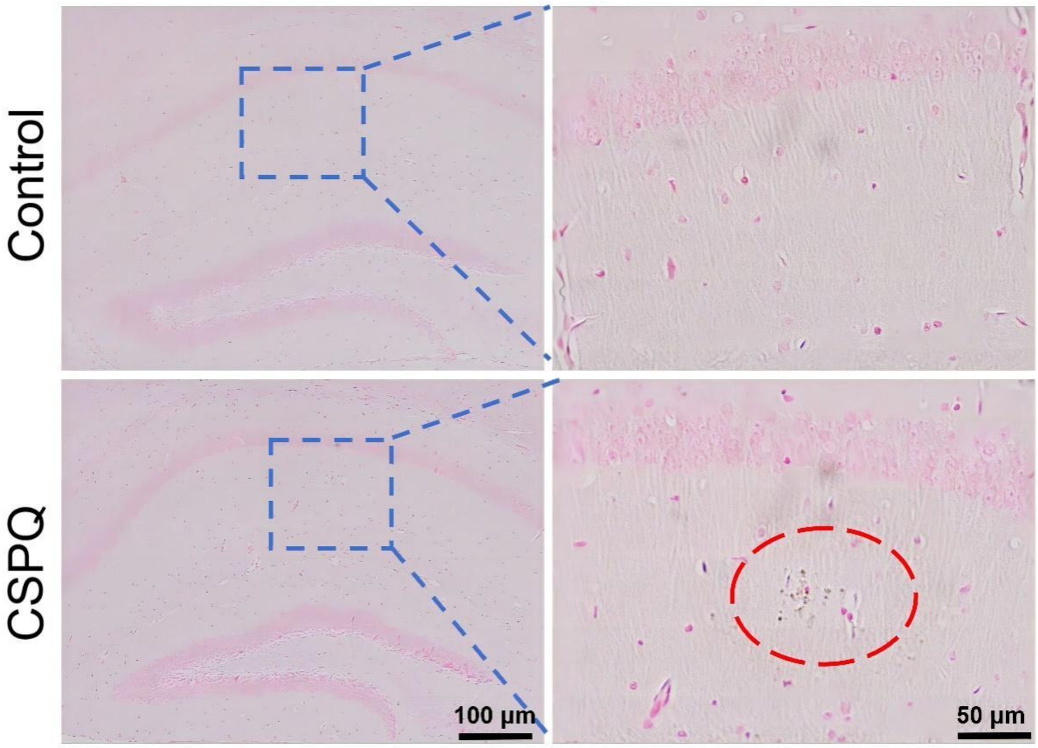

Figure S8. The deposition of CSPQ@CM nanoparticles in the brain determined by rubeanic acid (RA) staining. The experimental groups of mice were injected with the nanoparticles (dose: 12 mg/kg) through the tail vein after treatment with US. The healthy mice without any treatment were used as the control (n = 5 mice for each group, copper stains were indicated by the red circle, scale bar = 100 μm; and 50 μm in the enlarged insert).

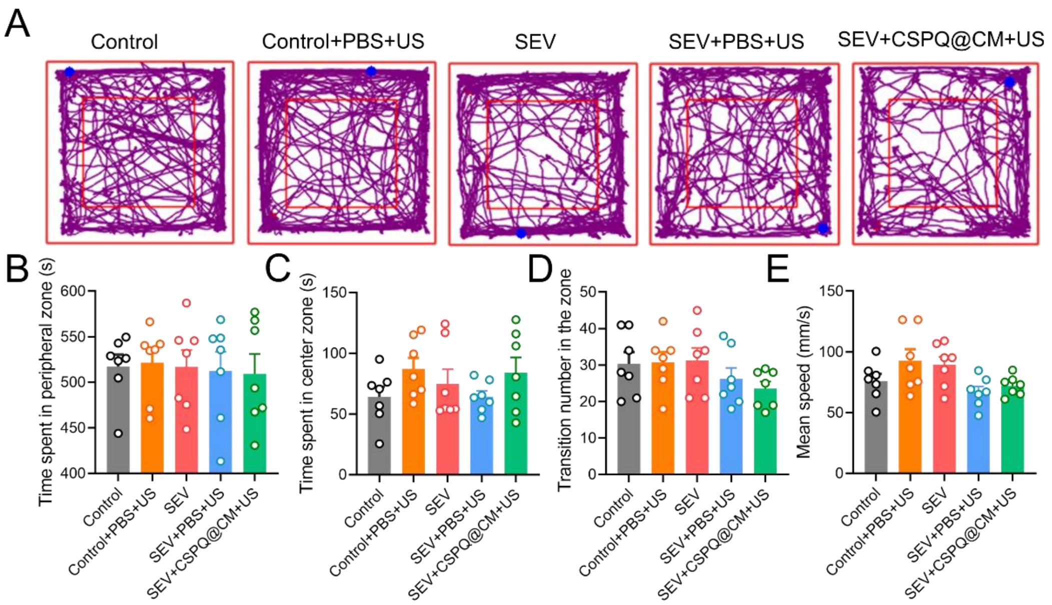

Figure S9. The effect of CSPQ@CM nanoparticles on the spontaneous locomotor activity of mice. (A) Representative motion track of mice obtained from the open field test (n = 7 mice for each group). (B) The time of mice spending in peripheral zone. (C) The time of mice spending in center zone. (D) The number of zone transition. (E) The mean speed from different groups of mice (n = 7 mice for each group). The two-sided one-way ANOVA with a Tukey post hoc analysis was used for comparison among multiple groups. Values were expressed as the mean ± SEM.

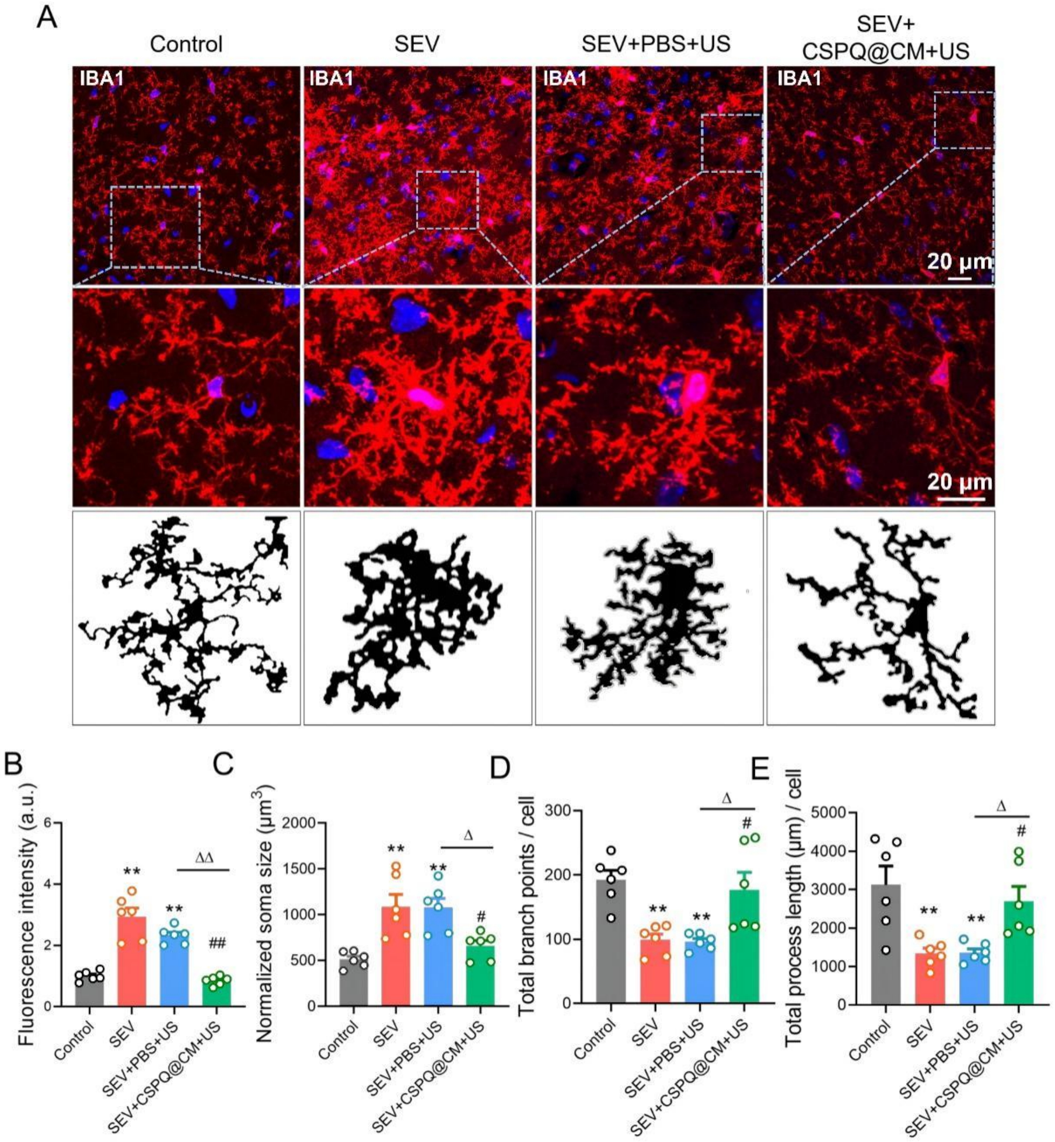

Figure S10. CSPQ@CM nanoparticles regulated the activation of microglia in mice caused by multiple sevoflurane exposures in their neonatal period. (A) Immunostaining images of microglial marker IBA-1 in the hippocampus (Scale bar = 20 μm). (B) The fluorescence intensity of IBA-1^+ microglia (n = 6 mice for each group). (C) The normalized soma size (n = 6 mice for each group). (D) The number of branch points of IBA-1^+ microglia (n = 6 mice for each group). (E) The total process length (n = 6 mice for each group). The two-sided one-way ANOVA with a Tukey post hoc analysis was used for comparison among multiple groups. Values were expressed as the mean ± SEM. $^*p < 0.05$, $^{**}p < 0.01$ *vs* Control group; $^{\#}p < 0.05$, $^{\#\#}p < 0.01$ *vs* SEV group; $^{\Delta}p < 0.05$ *vs* SEV+PBS+US group.
